# Metabolic activation and colitis pathogenesis is prevented by lymphotoxin β receptor expression in neutrophils

**DOI:** 10.1038/s41385-021-00378-7

**Published:** 2021-02-10

**Authors:** Thomas Riffelmacher, Daniel A. Giles, Sonja Zahner, Martina Dicker, Alexander Y. Andreyev, Sara McArdle, Tamara Perez-Jeldres, Esmé van der Gracht, Mallory Paynich Murray, Nadine Hartmann, Alexei V. Tumanov, Mitchell Kronenberg

**Affiliations:** 1 La Jolla Institute for Immunology, La Jolla, CA USA; 2 Kennedy Institute of Rheumatology, University of Oxford, Oxford, UK; 3 Department of Pharmacology, University of California San Diego, La Jolla, CA USA; 4 Department of Microbiology, Immunology and Molecular Genetics, University of Texas Health Science Center San Antonio, San Antonio, USA; 5 Division of Biological Sciences, University of California San Diego, La Jolla, CA USA

## Abstract

Inflammatory bowel disease is characterized by an exacerbated intestinal immune response, but the critical mechanisms regulating immune activation remain incompletely understood. We previously reported that the TNF-superfamily molecule TNFSF14 (LIGHT) is required for preventing severe disease in mouse models of colitis. In addition, deletion of lymphotoxin beta receptor (LTβR), which binds LIGHT, also led to aggravated colitis pathogenesis. Here, we aimed to determine the cell type(s) requiring LTβR and the mechanism critical for exacerbation of colitis. Specific deletion of LTβR in neutrophils (LTβR^ΔN^), but not in several other cell types, was sufficient to induce aggravated colitis and colonic neutrophil accumulation. Mechanistically, RNA-Seq analysis revealed LIGHT-induced suppression of cellular metabolism, and mitochondrial function, that was dependent on LTβR. Functional studies confirmed increased mitochondrial mass and activity, associated with excessive mitochondrial ROS production and elevated glycolysis at steady-state and during colitis. Targeting these metabolic changes rescued exacerbated disease severity. Our results demonstrate that LIGHT signals to LTβR on neutrophils to suppress metabolic activation and thereby prevents exacerbated immune pathogenesis during colitis.

## Introduction

Inflammatory bowel disease (IBD) affects over 1.5 million US citizens, and the incidence of disease is rising.^1^ While the pathogenesis of IBD is multifactorial, it is well established that the immune system plays a substantial role in the development of disease.^2^ Targeting specific immune mediators by biologics has become an increasingly widely used and effective treatment option.^3^ Among available biologics, antibodies specific for blockade of tumor necrosis factor (TNF) are currently the most commonly used therapy. However, only ~60% of patients treated with anti-TNF therapies show improvement,^3^ suggesting there are additional immune mediators critical to disease progression.^4^ While other cytokines, integrins, and molecules involved in immune signaling have been targeted with some success,^4^ the TNF superfamily of cytokines itself includes several other members that play important roles in modulating immune responses, suggesting they also could have potential to influence IBD development.^5^

Our previous work demonstrated that a member of the TNF superfamily, TNFSF14 or LIGHT (homologous to **l**ymphotoxins, exhibits **i**nducible expression, and competes with HSV **g**lycoprotein D for **H**VEM, a receptor expressed by **T** cells), plays a significant role in two mouse models of colitis. Unlike TNF, LIGHT expression was found to provide a protective effect in the T-cell transfer and dextran sodium sulfate (DSS) colitis models.^6^ This is surprising, because in models of lung and skin inflammation, LIGHT generally has been found to be pro-inflammatory.^7,8^ Although LIGHT binds to two receptors, the Herpes Virus Entry Mediator (HVEM or TNFRSF14) and the lymphotoxin β receptor (LTβR or TNFRSF3),^9^ we previously determined that only the LIGHT-LTβR interaction is critical for protection from severe DSS colitis.^10^ A similar protective role for the LTβR during DSS colitis was found by other groups.^11,12^

LTβR is expressed by a variety of structural and innate immune cells, including stromal cells, epithelial cells, macrophages, and neutrophils, but it is not typically expressed by lymphocytes.^9^ Of interest, each of these cell types has been previously shown to contribute to colitis pathogenesis. Fibroblasts are primary drivers of fibrosis in the intestine, a hallmark of colitis.^13^ Epithelial cell death and shedding of the epithelium correlates with more severe colitis.^14^ Macrophages play a prominent role in the pathogenesis of colitis.^15^ Although neutrophils contribute to intestinal inflammation, they also have been reported to contribute to disease resolution.^16^

LIGHT is part of a complex network of receptors and ligands, because each of its receptors, LTβR and HVEM, binds to proteins in addition to LIGHT.^9^ The LTβR also binds to lymphotoxin αβ hetero-trimers expressed on the cell surface.^5^ The interaction between these different receptors and ligands is complex, because removal of one interaction may affect interactions between others in the network.^17^ Here we carried out experiments to identify the cell type(s) in which LTβR expression is required and to identify the mechanisms downstream of the LIGHT–LTβR interaction that are involved in limiting severe disease. Our data indicate that neutrophil-specific loss of LTβR (LTβR^ΔN^) is sufficient to exacerbate DSS colitis. Mechanistically, LIGHT signaling through LTβR limited glycolysis, mitochondrial mass, mitochondrial respiration, and mitochondrial ROS production in neutrophils at baseline, suggesting a cell intrinsic mechanism. A similar metabolic change was also observed in colon neutrophils during DSS-induced colitis, and when targeted with the metabolic modulator metformin in LTβR^ΔN^ mice, exacerbated DSS-induced colitis was reversed. Thus, our data identify a novel function for LTβR in neutrophils, limiting mitochondrial metabolism and ROS, that contributes to preventing severe colitis.

## Results

### LTβR expression by neutrophils is protective

We previously identified that LTβR, not HVEM, is the relevant receptor for LIGHT-mediated protection from exacerbated colitis.^6,10^ LTβR expressing cell types in the colon lamina propria include macrophages, fibroblasts, epithelial cells, and neutrophils.^6,10^ To identify the disease-relevant LTβR-expressing cell type(s), we crossed mice with a floxed *Ltbr* allele to Cre mouse lines to delete receptor expression in specific cell lineages. Inducible deletion of the gene encoding LTβR in monocytes and macrophages was obtained in *Ltbr*^*fl/fl*^ × Cx3cr1-Cre ERT mice after tamoxifen treatment. The effect of fibroblast-specific deletion of LTβR was assessed via radiation chimeras that were *Ltbr*^*fl/fl*^ × Fsp1-Cre mouse recipients of wild-type (WT) bone marrow (BM). LTβR deletion in intestinal epithelial cells was achieved in *Ltbr*^*fl/fl*^ × Villin-Cre mice. Cx3cr1 is known to be expressed in mature intestinal macrophages^6^ and its expression is important for their homeostasis and the prevention of intestinal inflammation.^18^ We verified that Cx3cr1-Cre ERT caused ablation of LTβR expression specifically in monocytes from blood in response to tamoxifen (Supplementary Fig. 1a). The severity of colitis was not increased in these mice, based on weight loss, fibrotic colon shortening, histological scoring, and survival (Supplementary Fig. 1). Despite significant weight loss after day 10, histologic analysis indicated fairly mild inflammation in mice exposed to this DSS concentration. As reported previously,^19^ we found that Fsp1-Cre, when crossed to a fluorescent reporter mouse strain, also caused deletion in many hematopoietic cells (Supplementary Fig. 2a). Therefore, we analyzed BM chimeras in which WT BM was used to reconstitute *Ltbr*^*fl/fl*^ × Fsp1-Cre mice or *Ltbr*^*fl/fl*^ control mice. Severity of colitis was not increased when the donor BM was from Fsp1-Cre × *Ltbr*^*fl/fl*^ mice with relatively mild inflammation in this case as well (Supplementary Fig. 2). Deletion of the *Ltbr* gene in *Ltbr*^*fl/fl*^ × Villin-Cre mice was reported previously^20^ and we confirmed specific deletion in epithelial cells (Supplementary Fig. 3a). The severity of DSS-colitis compared to Cre-negative littermate controls was not increased in *Ltbr*^*fl/fl*^ × Villin-Cre mice and weight loss was in fact mildly improved (Supplementary Fig. 3).

**Fig. 1:**
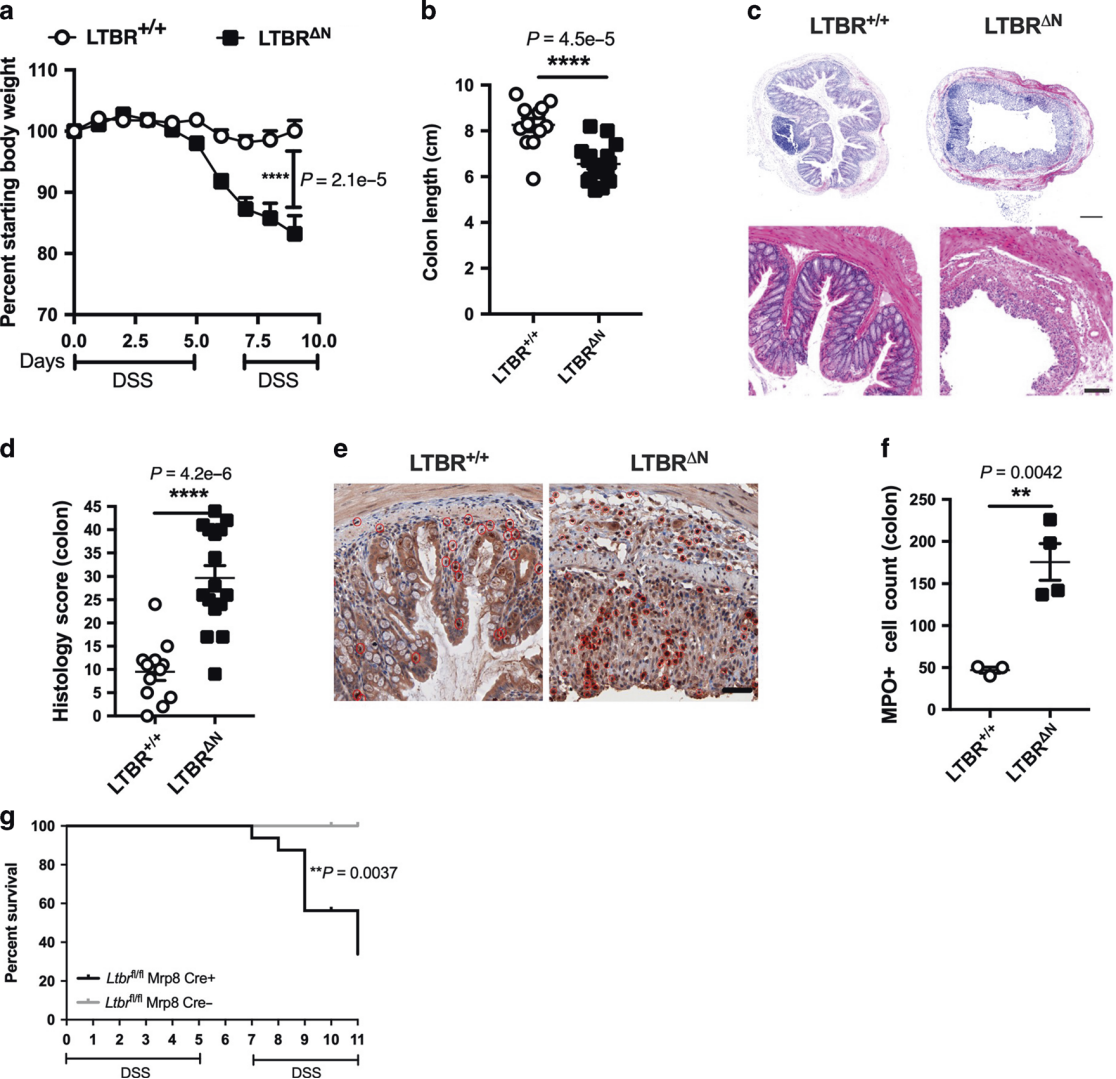
LTβR expression in neutrophils protects from DSS-colitis. Colitis was induced in *Ltbr*^*fl/fl*^ × Mrp8-Cre (LTβR^ΔN^) and control littermates by cycles of 2.5% DSS for 5 days followed by 2 days of water and disease progression was monitored. **a** weight loss was monitored daily as % starting body weight. **b** Colon length on day 9. **c** H&E stained representative histology sections of distal colon at 20× (top; scale bar, 200 μm) and 40× (bottom, scale bar, 100 μm) magnification. **d** Blinded total histological score from distal colon. Representative immunohistochemical MPO staining (**e**) and quantification (**f**) of MPO-positive neutrophils from sections of distal colon tissue. Scale bar, 50 μm **g** Kaplan–Meier survival plots of the indicated groups during two cycles of DSS. Data representative of >3 independent experiments (**a**, **c**, **e**-**g**) or pooled data from three experiments (**b**, **d**). Student’s *t* test (**b**, **d**, **f**), two-way ANOVA (**a**, **g**). Error bars represent SEM.

In contrast to those results, deletion of LTβR expression in neutrophils driven by *Ltbr*^*fl/fl*^ × Mrp8-Cre (LTβR^ΔN^; Supplementary Fig. 4a) was sufficient to induce accelerated weight loss (Fig. 1a) and colon shortening (Fig. 1b), as well as a greatly increased histological disease score (Fig. 1c, d). After DSS treatment, LTβR^ΔN^ mice showed a loss of epithelial integrity and mononuclear cell infiltration to the colon with edematous regions. These characteristics closely correlated with previous results in LIGHT-deficient mice.^6^ We confirmed that virtually all neutrophils express LTβR protein in WT mice at comparable levels with other myeloid cells, while no LTβR expression was found on B cells. About 10% of myeloid progenitor cells also expressed LTβR and deletion of LTβR by Mrp8-Cre was specific to neutrophils (Supplementary Fig. S4b). For this and following functional flow-cytometric experiments, neutrophils were gated based on CD11b+ and Ly6G+ expression (Supplementary Fig S4c, left), or magnetically enriched to about 90% purity, as indicated (Supplementary Fig. S4c, right). DSS-treated LTβR^ΔN^ mice exhibited a significant increase in Mpo staining, a marker for neutrophils, in the distal colon (Fig. 1e, f). Exacerbated DSS colitis was also evident in survival studies, as LTβR^ΔN^ mice had significantly increased mortality rates during the second cycle of DSS as compared to littermate controls (Fig. 1g). Together, these data suggest that LTβR expression by neutrophils is most important for protection of exacerbated DSS-induced colitis.

### Some neutrophil functions are normal in LTβR^ΔN^ mice

We analyzed cytokine production in colon fragment cultures at an early time during DSS treatment (day 5) and found no difference in LTβR^ΔN^ mice compared to littermate controls for the secretion of IL-6, IL-10, MCP-1, IFNγ, and TNF (Supplementary Fig. 5a). At later timepoints, cytokine levels were increased in LIGHT- or LTβR-deficient mice.^6,21^ To determine how the function of LTβR-deficient neutrophils might be directly altered at steady state and in response to bacterial triggers, we carried out a battery of neutrophil functional assays. Because obtaining the numbers of viable neutrophils required for functional assays was not possible from the intestines of DSS-treated mice, we measured the kinetics of *Escherichia coli* bioparticle phagocytosis by purified BM-derived neutrophils at steady state or after LPS-mediated activation and observed no effect of *Ltbr* deletion (Supplementary Fig. 5b). Further, despite the role of TNF-family receptors, including LTβR, in cell death signaling,^22,23^ we found no difference in the survival of neutrophils, purified from BM and cultured in vitro (Supplementary Fig. 5c). We then tracked neutrophils in vivo using a BrdU pulse-chase method at steady state and during DSS colitis. Labeled neutrophils appeared in the blood by day 4, then declined rapidly the next day, with no evidence for an early maturation or prolonged circulation in the absence of LTβR expression (Supplementary Fig. 5d). Neutrophil recruitment to the colon on day 7 of DSS treatment was comparable irrespective of LTβR expression as quantified by flow cytometry as the fraction of BrdU-positive neutrophil numbers in colon tissue (Supplementary Fig. 5e). To examine the effect of LTβR expression on neutrophils during an acute infection, we analyzed mice after pulmonary infection with *Streptococcus pneumoniae*, which requires neutrophil activity for clearance.^24^ The number of colony forming units (CFU) of *S. pneumoniae* in the lungs of control or LTβR^ΔN^ mice was similar, with a trend toward increased CFU (Supplementary Fig. 5f). This coincided with a 4–5-fold increase of neutrophils in the lung tissue 18 h post infection, which was mildly elevated in the absence of LTβR (Supplementary Fig. 5g). The robust, LTβR-independent recruitment and infiltration particularly of perivascular lung tissue was also evident from immunofluorescent Ly6G-analysis of tissue sections (Supplementary Fig. 5h). In conclusion, by these measures we did not find evidence for highly altered functioning of neutrophils from LTβR^ΔN^ mice at steady state or during acute bacterial infection in the lung.

### RNA-Seq reveals suppression of neutrophil metabolism by LIGHT-LTβR

**Fig. 2:**
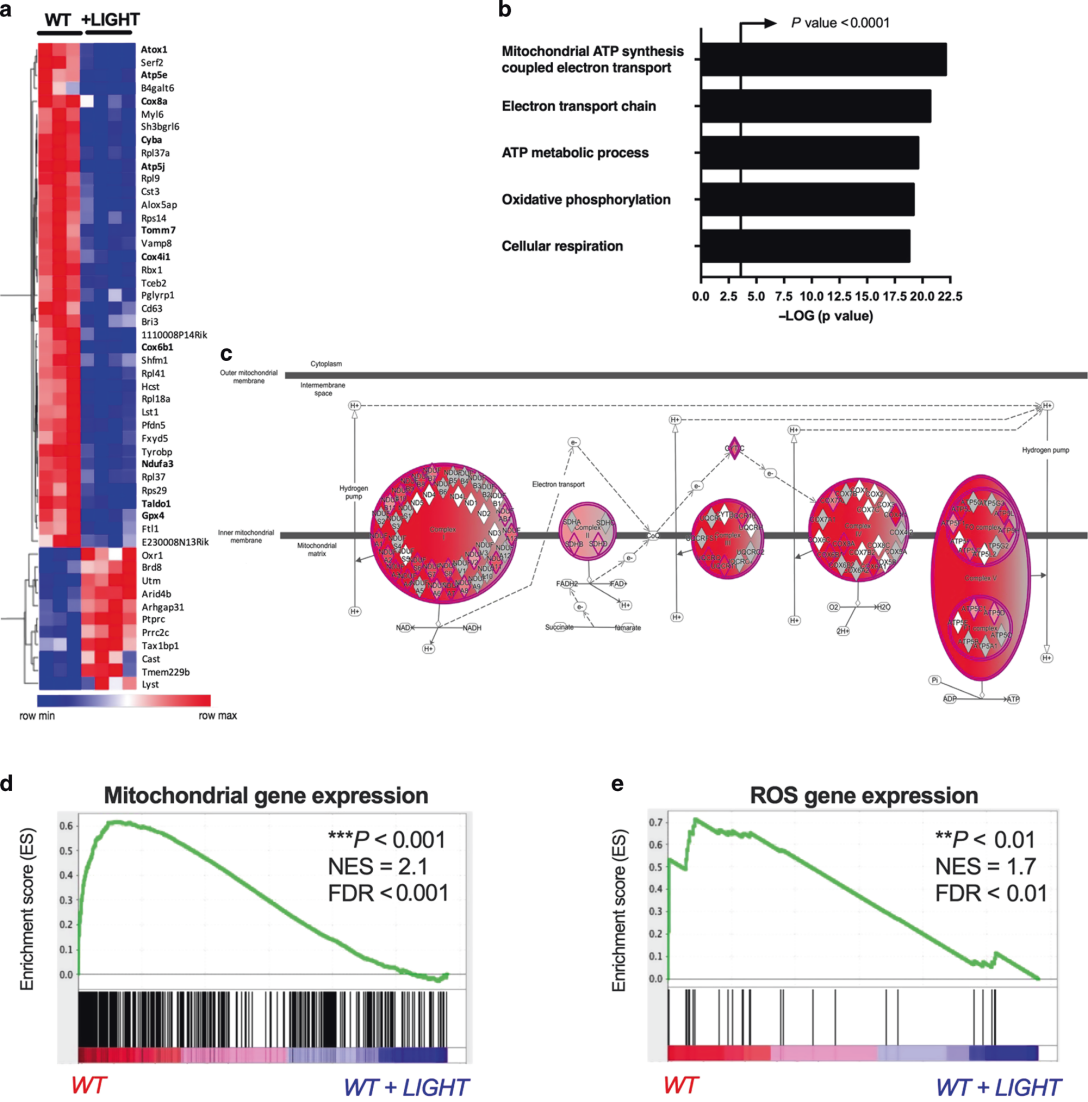
LIGHT signaling represses genes associated with mitochondrial function and ROS. RNA-Seq analysis of sorted blood neutrophils from WT mice stimulated with a DSS-associated cytokine cocktail and with or without recombinant mouse LIGHT. **a** The top 50 most differentially expressed genes with respect to *P* value. Genes in bold font are associated with mitochondrial and/or ROS pathways. **b** Gene expression pathways upregulated in the absence of LIGHT stimulation were determined by the ToppGene Suite. **c** Ingenuity Pathway Analysis (Qiagen) identified oxidative phosphorylation as a pathway highly upregulated in the absence of LIGHT stimulation. Significantly upregulated genes and complexes of the electron transport chain are depicted in red. Gray: not significantly different. White: not determined in dataset. **d**, **e** GSEA of the comparison of WT neutrophils and WT stimulated with LIGHT. **d** GSEA for genes associated with mitochondrial function from the molecular signature database list GO_mitochondrial_gene_expression. ***P < 0.001; NES = 2.1; FDR < 0.001. **e** GSEA for genes associated with cellular ROS pathways from the molecular signature database list Houstis_ROS. **P < 0.01; NES = 1.7; FDR < 0.01. GEO accession number GSE150243.

To identify which other LTβR-dependent pathways contributed to the more severe colitis, blood neutrophils were cultured with cytokines associated with more severe colitis in this model system, including IL-6, TNF, and GM-CSF,^6^ and in the presence or absence of recombinant, soluble mouse LIGHT protein. We carried out bulk RNA-Seq in these neutrophils and determined that soluble LIGHT stimulation significantly altered the expression of 458 genes, with a greater number of genes with reduced as opposed to increased expression by exposure to LIGHT. The top 50 differentially expressed genes by *P* value contained 11 mitochondrial- and/or ROS pathway genes, with all of these significantly decreased by LIGHT addition and none that were increased (Fig. 2a, Supplementary Table 1). In agreement with this, pathway analysis of all differentially expressed genes identified a significant enrichment for genes associated with mitochondrial biogenesis and the electron transport chain (ETC), which had increased expression in the absence of a LIGHT signal (Fig. 2b). Ingenuity analysis of the oxidative phosphorylation pathway confirmed that transcripts for multiple components of every complex within the ETC were significantly increased in the absence of LIGHT (Fig. 2c). Gene set enrichment analysis confirmed an increase in mitochondrial gene expression (Fig. 2d) and reactive oxygen species (ROS) gene sets when LIGHT was not added (Fig. 2e).

The suppressive effect of LIGHT was greatly reduced in LTβR-deficient neutrophils, despite neutrophil expression of HVEM, which also binds LIGHT. RNA-Seq analysis of LTβR-deficient neutrophils exposed to LIGHT showed increased expression of mitochondrial and ROS pathway genes compared to LIGHT-exposed LTβR WT neutrophils (Supplementary Fig. 6a, b). Transcripts of complex I, III, IV, and V subunits within the ETC were increased in LTβR-deficient neutrophils (Supplementary Fig. 6c), a subset of the genes that were also increased comparing WT neutrophils in the absence of LIGHT to LIGHT addition. Gene set enrichment analysis confirmed that gene ontology terms for mitochondrial genes and ROS pathway were increased in LTβR-deficient neutrophils (Supplementary Fig. 6d, e). Together, these data indicate that the LIGHT–LTβR interaction dampens expression of genes associated with mitochondrial function and ROS production within neutrophils exposed to inflammatory cytokines.

### LTβR affects neutrophil respiration and glycolysis

**Fig. 3:**
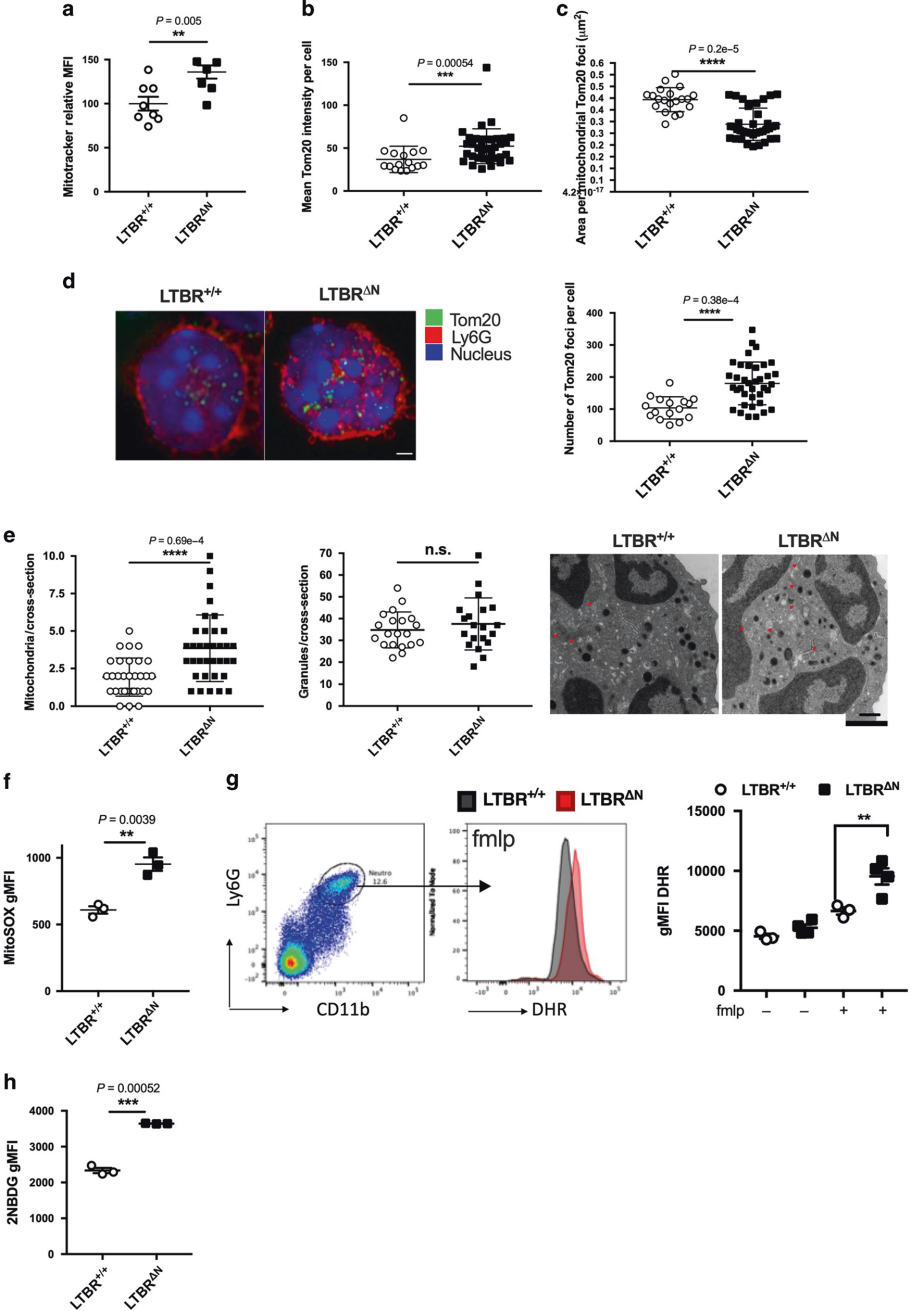
Energy-metabolic pathways are suppressed by LTβR. Neutrophils were detected from BM of *Ltbr*^ *fl/fl*^ × Mrp8-Cre (LTβR^ΔN^) and control littermates by flow cytometry (**a**, **f**, **g**) or by negative magnetic enrichment (STEMCCELL technologies) (**b–e**) and metabolic parameters were measured. Mitochondrial mass was measured as mean intensity of Mitotracker staining (**a**) and mean Tom20 immunofluorescence intensity by confocal microscopy (**b**). **c**, **d** Tom20 foci indicative of mitochondria were quantified from z-stacked confocal micrographs of Ly6G+ cells (red) stained with Tom20 antibody (green) and quantified by Imaris. Organization of Tom20 in clusters and the 3D-shape reflected in z-stacks can lead to multiple individual foci recorded for one mitochondrium. Scale bar, 500 nm **e** Representative TEM images (right) and blinded quantification of granule and mitochondrial content (left). Red arrows indicate individual mitochondria. Scale bar, 500 nm. **f** Neutrophil mitochondrial superoxides were measured by MitoSox and quantified by flow cytometry as mean fluorescence intensity. **g** Representative gating and histograms (left) and quantification (right) of neutrophil oxidative burst assay at baseline and in response to fMLP (fMLP N-formylmethionine-leucyl-phenylalanine; DHR dihydrorhodamine-123). **h** Fluorescent glucose uptake was quantified using the fluorescent glucose analog 2-NBDG (Invitrogen) by flow cytometry. Student’s *t* test, data from 3–6 mice/group from 2 or more independent experiments. Error bars represent SEM.

To determine if these transcriptional changes translate to functional differences in energy metabolism under steady-state conditions, we compared neutrophils from LTβR^ΔN^ mice to controls. Because of the need for relatively large numbers of viable cells, we analyzed neutrophils isolated from BM. Results from Mitotracker staining indicated that LTβR^ΔN^ BM neutrophils had higher levels of total mitochondrial mass (Fig. 3a). This technique does not differentiate between increased numbers of mitochondria or mitochondria of increased size. By using the parallel techniques of anti-Tom20 immunofluorescence and transmission electron microscopy (TEM) on magnetically sorted neutrophils, we found that LTβR-deficient neutrophils had significantly elevated numbers and mass of mitochondria per cell, but individual mitochondria had reduced surface area, (Fig. 3b–e). As an internal reference, no difference was detected in the number of neutrophil antimicrobial granules (Fig. 3e). Elongated mitochondrial networks are required for efficient ETC-mediated ATP production, while a small fragmented mitochondrial morphology points toward non-ATP-associated mitochondrial functions.^25^ In neutrophils, mitochondria can be dispensable for ATP balance, but central for ROS generation.^26^ Considering the association of neutrophil derived ROS with IBD,^21,27^ we determined if these changes in the mitochondrial compartment in LTβR^ΔN^ mice could be linked to ROS levels. Flow-cytometric analysis of neutrophils from LTβR^ΔN^ mice showed higher levels of mitochondrial superoxide levels than littermate controls, as measured by MitoSox, a ROS reporter that specifically localizes to mitochondria (Fig. 3f). Because mitochondrial and NADPH-oxidase dependent ROS production are connected, we next measured total ROS production in an oxidative burst assay. Within 30 min after stimulation with N-formylmethionine-leucyl-phenylalanine (fMLP), fluorescence of the total ROS-indicator dihydrorhodamine-123 (DHR) was induced by oxidation. The induced total ROS response detected was significantly elevated in neutrophils from LTβR^ΔN^ mice compared to controls (Fig. 3g).

In addition to pro-inflammatory activity,^21^ ROS production and elevated mitochondrial activity can lead to increased aerobic glycolysis, a pathway central to most neutrophil functions.^28^ As a surrogate for glycolysis we therefore measured the consumption of 2-NBDG, a fluorescent glucose analog. LTβR-deficient BM neutrophils exhibited greatly increased 2-NBDG accumulation, indicating increased glucose uptake (Fig. 3h).

RNA-Seq data demonstrated that mitochondrial metabolism and the ROS response were transcriptionally downregulated in neutrophils by soluble LIGHT, but this required LTβR expression. Consistent with data indicating that LIGHT–LTβR prevented severe colitis, we hypothesized that the metabolic changes in neutrophils would be linked to colitis severity and, therefore, changes in metabolic activity in LTβR-deficient neutrophils would also be present in neutrophils from LIGHT-deficient mice. Indeed, neutrophils isolated from *Light*^*−/−*^ animals showed increased mitochondrial ROS and glycolysis, while mitochondrial mass remained unchanged (Supplementary Fig. 7a–c). Overall, the data support the assertion that LIGHT stimulation of LTβR suppresses neutrophil metabolic activity.

### Metabolic flux measurements of LTβR-deficient neutrophils

**Fig. 4:**
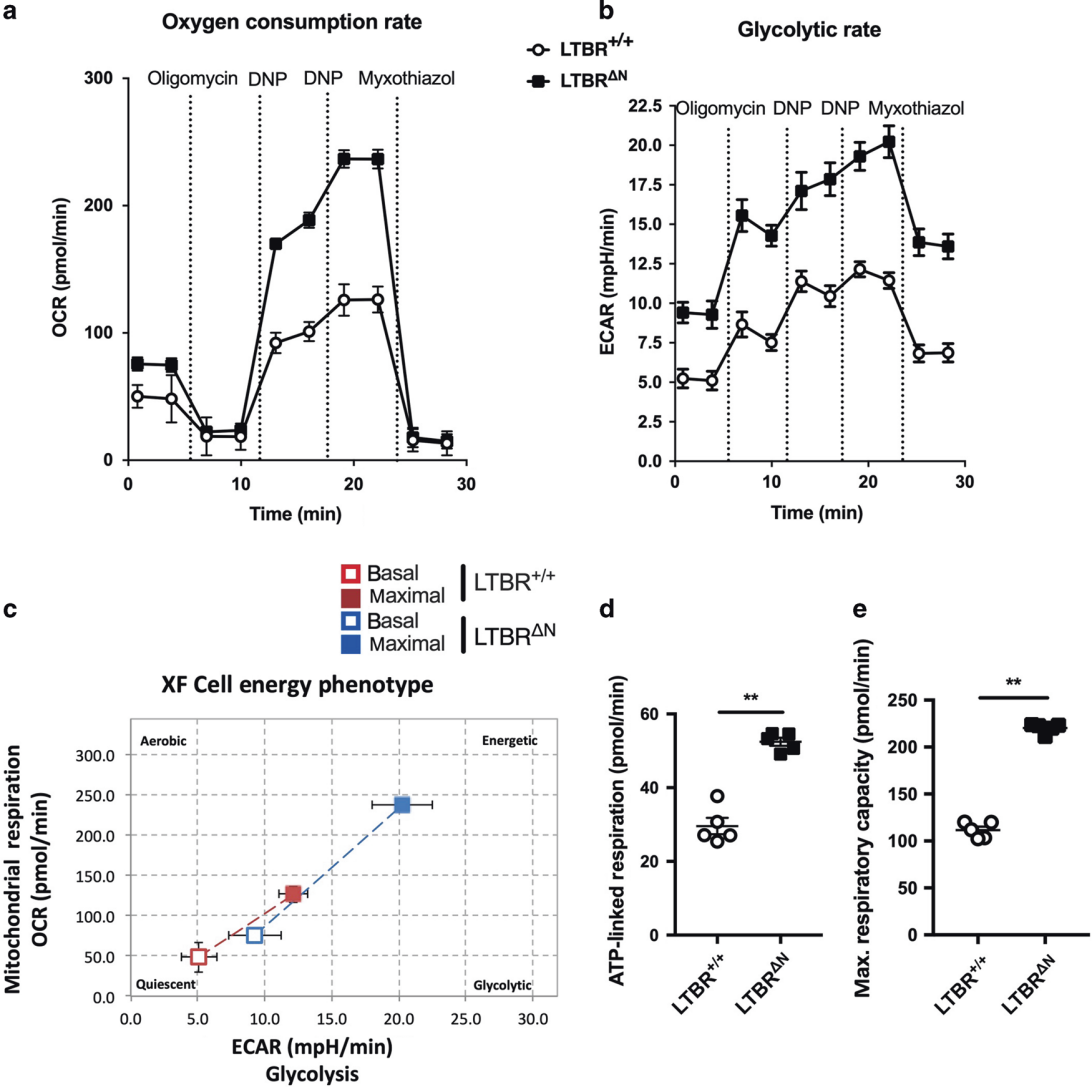
LTβR dampens neutrophil respiration and glycolysis. CD11b+ Ly6G+ Neutrophils were isolated from BM of *Ltbr*^*fl/fl*^ × Mrp8-Cre (LTβR^ΔN^) and control littermates (pooled from 5 mice/group) by magnetic sorting and their metabolic parameters were measured. **a** Oxygen consumption rate (OCR) and **b** extracellular acidification rate (ECAR) were measured in real time at baseline and in response to the indicated mitochondrial inhibitors. See “Methods” for details. **c** Basal and maximal mitochondrial respiration and glycolysis are mapped to illustrate pathway preference. ATP-linked respiration (**d**) and maximal respiratory capacity (**e**) are quantified as ΔOCR from flux analysis. Data from 5 mice/group, measured as mean of 5 wells for each timepoint, representing one of three independent experiments. Error bars represent SEM.

We next measured real-time rates of mitochondrial respiration and extracellular acidification, indicative of glycolytic flux, in BM neutrophils from LTβR^ΔN^ mice and littermate controls using the Seahorse bio-analyzer. This revealed a twofold increase in the basal respiration rate and a proportional increase in spare mitochondrial respiration capacity, as measured by the oxygen consumption rate (OCR) following DNP in BM neutrophils from LTβR^ΔN^ mice (Fig. 4a). In addition, there was a significant, twofold increase in the extracellular acidification rate (ECAR) of LTβR-deficient neutrophils (Fig. 4b). Elevated acidification may partially derive from increased CO_2_ production during oxidative phosphorylation, and from glycolysis-associated acidification. On a metabolic phenotype map, LTβR-deficient neutrophils had a proportionally higher basal- and maximal energetic profile, indicating strongly increased mitochondrial energy demand, sustained by elevated glycolytic flux, rather than a qualitative metabolic switch, in LTβR^ΔN^ mice (Fig. 4c). The heightened oxidative metabolism was also reflected in significantly increased ATP-linked respiration as quantified by ΔOCR post oligomycin treatment as well as profoundly increased maximal respiratory capacity (Fig. 4d, e). Despite these changes in cellular respiration, the total content of neutral lipids was unchanged (Supplementary Fig. 5i). Together, these data suggest that the lack of LTβR expression by neutrophils profoundly affected their metabolic programming at steady state and resulted in increased mitochondrial number, oxidative phosphorylation, mitochondrial ROS production and glycolysis at steady state.

### LTβR-dependent changes in metabolism persist in colon neutrophils

**Fig. 5:**
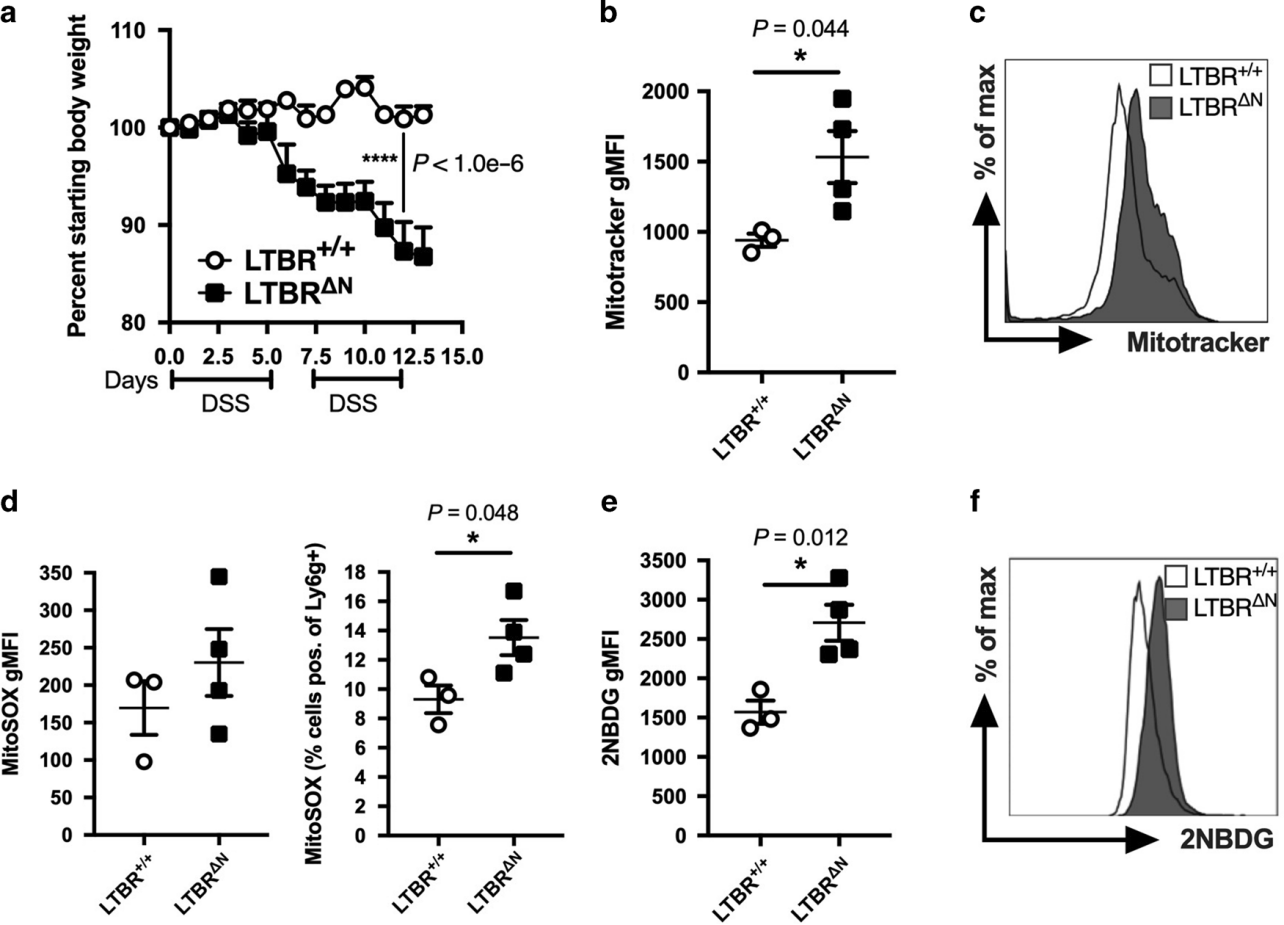
Colon inflammation is associated with metabolic neutrophil activation in the absence of LTβR. Colitis was induced in *Ltbr*^ *fl/fl*^ × Mrp8-Cre (LTβR^ΔN^) and control littermates by 2.5% DSS and disease and metabolic parameters were measured in colonic neutrophils. **a** Relative weight loss expressed as % of starting body weight. Quantification of neutrophil Mitotracker mean fluorescence intensity by flow cytometry (**b**) and representative histograms of colon neutrophil mitochondrial mass (**c**). **d** Mitochondrial superoxide production quantified by MitoSox by mean fluorescence intensity (left) and percent positive cells (right). Fluorescent glucose 2-NBDG uptake in neutrophils isolated from the colon quantified by flow cytometry (**e**) and representative histogram of 2-NBDG uptake (**f**). Student’s *t* test. Data from 3–4 mice/group, representative of 3 experiments. Error bars represent SEM.

We initially identified metabolic changes in LTβR-deficient neutrophils due to LIGHT–LTβR interactions based on the unbiased approach of RNA-Seq analysis. In that experiment, neutrophils from blood were cultured with cytokines in vitro to mimic the inflammatory state in DSS colitis. Therefore, we examined if the metabolic changes observed in ex vivo analysis of BM neutrophils were also present in colonic neutrophils of DSS-treated mice. LTβR^ΔN^ mice had accelerated weight loss that persisted in the chronic phase (Fig. 5a) and this was associated with increased colonic neutrophil mitochondrial mass (Fig. 5b, c), elevated mitochondrial ROS (Fig. 5d) and increased glucose uptake (Fig. 5e, f) compared to neutrophils from DSS-treated control mice colons.

### LTβR-dependent changes in metabolism are linked to severe colitis

**Fig. 6:**
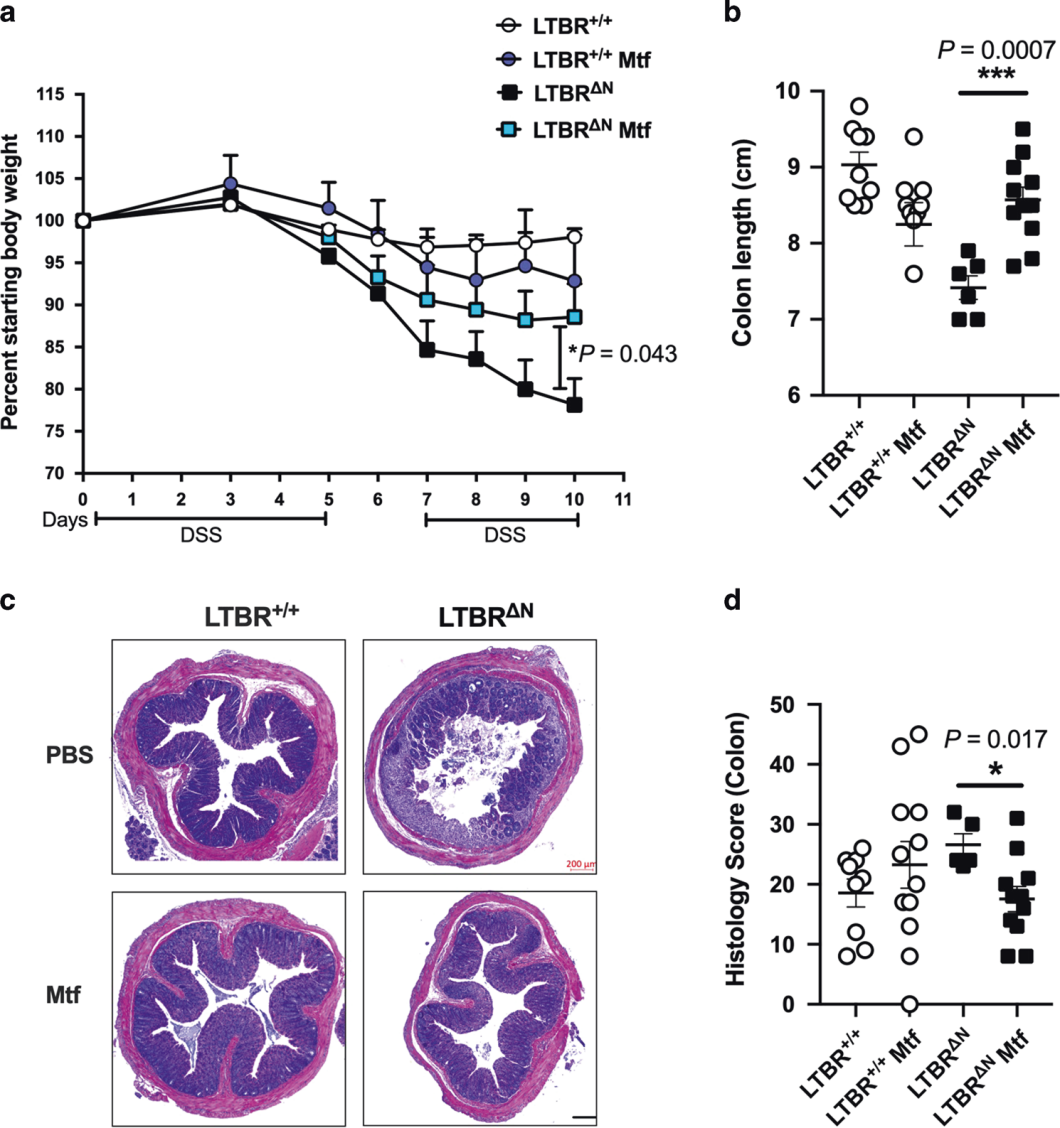
LTβR driven neutrophil metabolic activation causes exacerbated DSS-induced colitis. Colitis was induced in *Ltbr*^* fl/fl*^ × Mrp8-Cre (LTβR^ΔN^) and control littermates by 2.5% DSS. Metformin (Mtf, 200 mg/kg) or PBS was administered every other day by oral gavage and disease parameters were measured over time. **a** Weight loss was monitored daily as % starting body weight. **b** Colon length at endpoint of experiment. **c** H&E stained representative sections from distal colon with (**d**) blinded total histological score. Data represents three independent experiments (**a**, **c**) or is combined from three independent experiments (**b**, **d**). Significance tested by two-way ANOVA (**a**) or student’s *t* test (**b**, **d**). Scale bar, 200 μm. Error bars represent SEM.

ROS is a well characterized driver of colitis pathogenesis,^21,27^ and therefore we targeted ROS production in vivo. An earlier publication showed DSS-induced disease was altered in WT mice injected with the redox protective agent N-acetyl-cysteine (NAC) during colitis induction.^29^ Mice underwent daily i.p. administration of NAC during colitis induction, and although there was a trend toward reduced colitis in LTβR^ΔN^ mice, especially regarding colon shortening due to fibrosis, the difference did not reach statistical significance (Supplementary Fig. 8a, b). We did not find evidence, however, that the treatment affected ROS production when analyzed in colon neutrophils ex vivo. We therefore also tested metformin, which has several relevant actions: It reduces endogenous ROS through its action as a mild complex I mitochondrial inhibitor,^30^ acts as a NADPH-oxidase inhibitor and it reduces intestinal glucose availability.^31^ Due to this pleiotropic nature of metformin, it is not possible to definitively isolate individual contributions of these actions, while cooperative effects of the individual mechanisms of action on neutrophil metabolic state may benefit effect size. Metformin treatment was able to reverse the accelerated disease in LTβR^ΔN^ mice, including positive effects on weight loss (Fig. 6a), protection from fibrotic shortening of the colon (Fig. 6b), and it provided a significant improvement of the total disease score from colon histology (Fig. 6c, d). Together, these data suggest that metabolic dysregulation, including enhanced oxidative and glycolytic metabolism and ROS production in mice with neutrophil-specific LTβR deletion, persisted in the colon during DSS colitis and contributed to exacerbated disease.

## Discussion

LIGHT binds to HVEM and LTβR, but each receptor has binding partners in addition to LIGHT.^9^ Despite this possible source of redundancy, the LIGHT–LTβR interaction has unique functions in contributing to intestinal barrier dysfunction, causing fibrosis, and contributing to multi-organ inflammation when Light is overexpressed in T cells.^8,32–34^ In contrast to these pro-inflammatory functions, our previous work showed that the LIGHT–LTβR interaction prevented severe colitis induced by T-cell transfer or DSS.^6,10^ Therefore, the outcome of LIGHT signaling to the LTβR is highly context and cell type specific. In this study, we aimed to determine which LTβR expressing cell types were critical for providing protection from severe colitis, and which mechanism(s) downstream of the LIGHT–LTβR interaction were affording this protection.

Here we report three novel findings. First, we determined that neutrophil LTβR expression was important for preventing the exacerbation of inflammation in the colon induced by DSS. Second, we showed that LTβR expression in BM neutrophils regulates their metabolism at steady state, affecting several pathways including ROS production. Third, we showed that these differences in metabolism are also present in colonic neutrophils after DSS treatment, and our data linked the exacerbated inflammation resulting from the absence of LIGHT–LTβR signaling to altered mitochondrial metabolism and ROS generation.

Our data on a protective role for LTβR signaling in DSS colitis is partially consistent with other reports.^12,35^ Here, we provided novel data that neutrophils are the important cell type. The previous work implicated macrophages, based on the analysis of LysM-Cre mediated deletion^35^ and the observation that few neutrophils express LTβR.^35^ In contrast, we found previously that colonic neutrophils in DSS-treated mice had more *Ltbr* mRNA compared to monocytes and other colon cell types,^6^ and here we demonstrated that all BM neutrophils expressed surface LTβR. Furthermore, LysM-Cre deletes genes in neutrophils as well as macrophages. Moreover, although mature lamina propria macrophages express Cx3cr1,^18^ the results comparing *Ltbr* deletion using Mrp8-Cre to Cx3cr1-Cre ERT were striking in showing that only the neutrophil-specific gene deletion augmented colitis severity.

Increased levels of activated neutrophils in the colon are a hallmark of active IBD,^16^ and neutrophil proteins calprotectin, lipocalin, and myeloperoxidase (Mpo) are used as disease biomarkers.^36^ We found increased neutrophils in the colon of LTβR^ΔN^ mice, but we did not find evidence for increased generation and mobilization of neutrophils at steady state or homing to the intestine in DSS-treated mice. These data suggest altered homing was not the cause of worsened disease. Because of the balance of beneficial and pathologic impacts of neutrophils on colitis,^16^ however, we have not carried out neutrophil depletion. In fact, most mouse studies agree that neutrophil depletion aggravated disease,^37^ whereas modulation of neutrophil effector functions has been successfully employed in multiple pre-clinical studies.^16^

While our previous data indicated that abrogated LIGHT–LTβR interactions are responsible for severe disease,^6,10^ a previous study found that the absence of LIGHT was protective^38^ and that the absence of LTβ was the relevant ligand for increased colitis when it was missing from T cells.^35^ Although the previous work analyzed an acute DSS-induced model, in our experiments, deficiency for LIGHT accelerated disease in both the acute and chronic models.^6^ Likely this discrepancy may be due to microflora or other differences in mouse colonies. We note that LIGHT deficiency increased colitis even in DSS-treated *Rag*^*−/−*^ mice and therefore LIGHT was acting not in T cells but in the innate immune system.^6^ Colonic neutrophils expressed high amounts of both LIGHT and LTβR mRNA,^6^ suggesting a possible autocrine signaling loop mediated by LIGHT, although the cell type providing the required LIGHT remains to be identified. LTβR-dependent effects of LTβ in moderating severe colitis due to expression by T cells are not ruled out by our work, however, and overall the data indicate how the analysis of cell-type-specific effects of gene deficiency are essential in this complex system of ligands and receptors.

Regarding the second novel point, our findings support the hypothesis that LIGHT–LTβR signals affect BM neutrophil metabolism at steady state. The data suggest that the need for the LTβR signal was not confined to a discrete point during neutrophil differentiation, however, because blocking LTβR during disease onset also increased colitis pathogenesis.^6,39^ Furthermore, addition of LIGHT to WT blood neutrophils in vitro that were exposed to inflammatory cytokines dampened their metabolic response. This steady state effect of LTβR may be analogous to its effect on the organization of B and T lymphocytes in the spleen, where continual LTβR signaling is required.^32^ It remains to be explored which signaling pathways mediate the effect of LTβR on neutrophil energy metabolism. One factor might be TRAF3, which is known to participate in LTβR signaling.^33^ Deletion of TRAF3 in B cells phenocopied the activated mitochondrial phenotype and increased glucose uptake we observed in LTβR-deficient neutrophils.^34^ Regardless, while at steady state LTβR-deficient neutrophils have a higher metabolism, this is increased further when activated in several different ways, including exposure to fMLP and by exposure to inflammatory cytokines, which induces transcriptional activation of genes in this pathway. We propose that whether this contributes to inflammation, however, depends on other signals present in the tissue milieu.

Neutrophils are mostly reliant on glycolytic pathways, but energy metabolism is highly regulated during granulopoiesis and requires robust mitochondrial oxidative metabolism in progenitor cells.^40^ In line with this, aspects of the mitochondrial, oxidative neutrophil phenotype we describe are also found in c-kit^+^ neutrophils.^41^ As mature cells, when neutrophils encounter microbes, their effector response requires large ATP quantities sustained by glycolysis,^28^ but several recent reports also describe essential roles of mitochondria in the neutrophil response,^42–44^ including ROS production.^26,43^ Neutrophils more reliant on oxidative metabolism that produce ROS in the tumor microenvironment also have been described,^41^ and the dysregulation we observed in neutrophils from LTβR^ΔN^ mice may be similar to the aberrant neutrophil phenotype in the tumor microenvironment.

Consistent with the importance of LIGHT–LTβR interactions, we found evidence for an effect of LIGHT on energy metabolism in neutrophils by adding LIGHT in vitro or by analyzing cells from LIGHT-deficient mice. The effect in LIGHT-deficient mice was less pronounced than in LTβR^ΔN^ mice, suggesting the germline deletion of LIGHT could have compensating effects in different cell types. For example, LIGHT was reported to protect against diet-induced obesity, glucose intolerance, and insulin resistance^45^ and regulate lipase expression and lipid homeostasis by interacting with LTβR on cells other than neutrophils.^46,47^

With respect to the third novel point, our data suggest that the exacerbated colitis in the absence of LIGHT–LTβR signaling in neutrophils was caused in part by metabolic changes, especially increased ROS generation. Multiple lines of evidence from this and other studies support this conclusion, including the LIGHT–LTβR dependence of expression of genes related to metabolism, the increased metabolic function of LTβR-deficient neutrophils at steady-state and in the colon during colitis, and their enhanced response to signals such as fMLP. Other studies reported beneficial effects for NAC in colitis^29,48^ or have used metformin to reduce endogenous ROS^31^ and ameliorate DSS colitis.^49,50^ While our data using NAC did not quite reach significance, and we did not observe an effect of Metformin in WT mice, lowering mitochondrial and NADPH-dependent ROS output and glucose availability with metformin effectively blocked the severe disease phenotype of LTβR^ΔN^ mice. Therefore, taken together, these data support, although they do not prove, the hypothesis that altered neutrophil metabolism contributes to increased inflammation in the intestine. Because of the different metabolic effects of Metformin, however, it remains uncertain if increased ROS is the sole or main contributor to altered neutrophil function in the absence of LTβR.

Dysregulation of a proposed LTβR-mediated “metabolic brake” may be particularly consequential in the colon. Ample literature exists implicating reactive oxygen and nitrogen species in the pathogenesis of IBD,^51–53^ but therapeutic targeting with various antioxidants has only shown limited success clinically.^54^ This may be explained in part by the fact that ROS is also essential to support an efficient innate immune response which protects from colitis susceptibility.^55^ In fact, a reduced amount of ROS production is likely a causal factor in some pediatric IBD patients, and linked to alterations in genes that regulate glycolytic metabolism.^27^ Up to 40% of chronic granulomatous disease patients develop colitis, characterized by inadequate NADPH-oxidase mediated and mitochondrial ROS generation^56^ within phagocytes.^55^

The energetically dysregulated phenotype and heightened ROS from LTβR-deficient neutrophils, resulting in more severe inflammation, could result in a feed-forward loop that exacerbates the DSS-induced colitis. This could explain the increased colonic synthesis of mRNA for chemokines such as CXCL1 and CXCL2 and the increased neutrophil accumulation observed.^6^ We did not detect increased inflammatory IL-6 or TNF production in colonic fragment cultures, which may reflect the early timing of the assay. By no means do we exclude other LTβR-dependent effects on neutrophil functions contributing to colitis, aside from metabolic changes, that were previously reported to be dependent on LTβR signaling.^5,9^ We also note that neutrophil survival may be altered in the absence of LTβR signals in the colon, suggested by the increase in these cells after DSS treatment. At steady state using cultured BM cells, however, there was no difference in the survival of LTβR-deficient neutrophils.

The relevance of our findings for IBD patients remain to be determined. DSS may induce mucosal changes more similar to acute injury than colitis. In knockouts of the LTβR ligand LIGHT, however, we found increased disease and a similar activation of the innate immune response in both the DSS and T-cell transfer models. A region on chromosome 12, including *CD27*, *TNFRSF1A*, and *LTBR*, is associated with Crohn’s disease risk.^57^ Recently, a transcriptomic signature of neutrophil activation has been associated with poor outcome in a study of UC patients.^58^ Our data show that the LIGHT–LTβR signaling network requires cell-type specific fine-tuning to prevent inflammatory disease. This is relevant to currently ongoing clinical trials that use decoy receptor 3 fusion proteins, which block LIGHT–LTβR along with other TNFSF–TNFRSF interactions,^59^ as well as a LIGHT-targeting antibody tested for the treatment of IBD^60^ (phase 1b, NCT03169894).

## Material and methods

### Animals

All mice were bred and housed under specific pathogen-free conditions at the La Jolla Institute for Immunology (La Jolla, CA). All mice were on the C57BL/6J background. All Cre-recombinase expressing mice were from the Jackson Laboratories; Bar Harbor, ME. LTβR^*Δ*N^ mice were generated by crossing B6.Cg-Tg(S100A8-cre-EGFP)1Ilw/J (Mrp8-Cre) strain mice to *Ltrb*^*fl*/*fl*^ mice.^20^ Deletion of *Ltbr* in other cell lineages was done by crossing *Ltrb*^*fl*/*fl*^ mice either to tamoxifen-inducible Cx3cr1-Cre ERT (B6.129P2(C)-*Cx3cr1*^*tm2.1(cre/ERT2)Jung*^/J), Fsp1-Cre (B6.C-Tg(S100a4-cre)1Egn/JhrsJ), or Villin-Cre (B6.Cg-Tg(Vil1-cre)997Gum/J) expressing mice. To prevent hematopoietic off-target deletion by Fsp1-Cre, BM chimeras were generated by grafting sub-lethally irradiated *Ltbr*^ *fl/fl*^ × Fsp1-Cre mice with 1 × 10^7^ cells of WT BM, to achieve fibroblast-specific deletion. To analyze monocyte- and macrophage-specific conditional deletion, *Ltbr*^ *fl/fl*^ × Cx3cr1-Cre ERT mice were treated with 1 mg tamoxifen twice before the start of DSS treatment and tamoxifen administration was continued during DSS treatment. LIGHT-deficient mice (*Tnfsf14*^−/−^) were provided by Dr. Klaus Pfeffer (University of Düsseldorf, Germany).^61^ All procedures were approved by the La Jolla Institute for Immunology Animal Care and Use Committee and are compliant with the ARRIVE standards. Mice were littermates and animals in all experiments were age and sex matched. Mice of both sexes were analyzed, and mice were sex matched for individual experiments.

### Chronic DSS-induced colitis

Mice received 2.5% DSS (Affymetrix) in the drinking water for a maximum of three cycles. As previously described, one cycle is comprised of 5 days of water plus DSS and 2 days with regular drinking water.^10^ Animals in all experiments were age and sex matched. Mice of both sexes were analyzed, but mice were sex matched for individual experiments. Body weight and appearance were monitored daily. Mice were euthanized, in compliance with approved animal protocols, within 24 h of losing more than 20% of their starting body weight.

### Histology and immunohistochemistry

Following measurement of colon length, a piece of distal colon and cecum were fixed in zinc formalin (Medical Chemical Corporation). Following paraffin embedding, fixed tissue was stained with H&E. For Mpo immunohistochemistry staining, anti-Mpo antibody (ab9535, Abcam) was used. Images were generated from 5 or more sections per organ on an Axioscan-Z1 platform (Zeiss) using Zen-2.3 software. Slides were scored according to previously described criteria^6^ by a pathologist blinded to the experimental condition.

### Flow cytometry and antibodies

Fluorochrome-conjugated monoclonal antibodies and metabolic dyes and staining conditions are listed in Supplementary Material. Data were acquired using Fortessa or LSR II flow cytometers (BD Biosciences). Metabolic marker fluorescence intensity depends on the instrument type and laser intensity, and therefore does not allow inter-experiment comparisons.

### Metabolic flux analysis

The real-time OCR and ECAR were measured using an XFe96 extracellular flux analyzer (Seahorse Bioscience). For detailed conditions see Supplementary Material.

### Phagocytosis assay

Neutrophil phagocytosis was assessed by flow-cytometric quantification of fluorescent bead uptake during 30 min incubation in the presence or absence of 1 μg/ml LPS. pH-sensitive phRodo bioparticle-beads (Invitrogen) were used according to the manufacturer’s protocol and surface marker staining was performed after phagocytosis. Data recorded as percent of neutrophils with quantifiable levels of bead uptake.

### Bacterial infections

*S. pneumoniae* URF918 strain was cultured at 37 °C in an incubator with 5% CO_2_. Bacteria were grown in Todd Hewitt broth (BD Biosciences). During mid-log phase, bacteria were harvested, washed twice in phosphate-buffered saline (PBS) and 1–5 × 10^6^ CFU, in a volume of 45–50 μl PBS, were used for retropharyngeal infection. Lungs were harvested 2 dpi, homogenized, and spread on blood agar plates for determination of CFU.

### Microscopy (TEM, confocal)

For TEM, 1 × 10^6^ purified neutrophils were fixed in 2% glutaraldehyde in 0.1 M sodium cacodylate buffer and processed according to the University of California San Diego EM core protocol. For confocal microscopy, 1 × 10^6^ purified neutrophils were stained with antibodies as described for flow cytometry, and fixed in 2% para-formaldehyde for 30 min. Tom20 (D8T4N, Cell Signaling Technologies) antibody was used for detection of mitochondria. Cells were cytospun on glass coverslips and mounted in the presence of DAPI. Images were acquired on a ZEISS LSM 880 inverted confocal microscope with a 63×/1.46 NA plan-apochromat objective. The Airyscan module was used to improve resolution and signal-to-noise ratio. Automated image quantification was performed in Imaris (Bitplane) using the spot detection algorithm.

### Colon explant cytokine quantification

For cytokine explants, four 3 mm punch biopsies were obtained from distal colon at the end of the first DSS cycle (day 5). Biopsies were cultured in 48-well plates with 500 μL RPMI containing 10% FBS for 6 h. Supernatants were collected and analyzed by ELISA (Thermo Fisher) according to the manufacturer’s protocol.

### RNA-Seq

Details of RNA-Seq data acquisition and analysis are described in Supplementary Material. The raw fastq files have been submitted to the Gene Expression Omnibus under accession number GSE150243.

### Statistical methods

Data are plotted as mean ± standard error of the mean (SEM), and statistical significance was determined by using the Mann–Whitney *U* test or unpaired *t* test. Significance for multiparameter comparisons was determined by two-way ANOVA.

## Supplementary information

Supplementary File

## Data Availability

All flow cytometry analysis was conducted using FlowJo 10.4.2 software (FlowJo, Ashland, OR). All graphs and statistical analysis were generated using Prism 8 software (GraphPad Software, San Diego, CA). Automated quantifications were generated using Imaris (Bitplane).
