# Supplementary material for: Metabolic activation and colitis pathogenesis is prevented by lymphotoxin β receptor expression in neutrophils
Source: Mucosal Immunol. 2021 Feb 10;14(3):679–90. doi: 10.1038/s41385-021-00378-7 (PMC8075978; doi:10.1038/s41385-021-00378-7)
Supplement: Supplementary file 1 — Supplementary File [file 41385_2021_378_MOESM1_ESM.pdf]

# Supplemental Figure 1

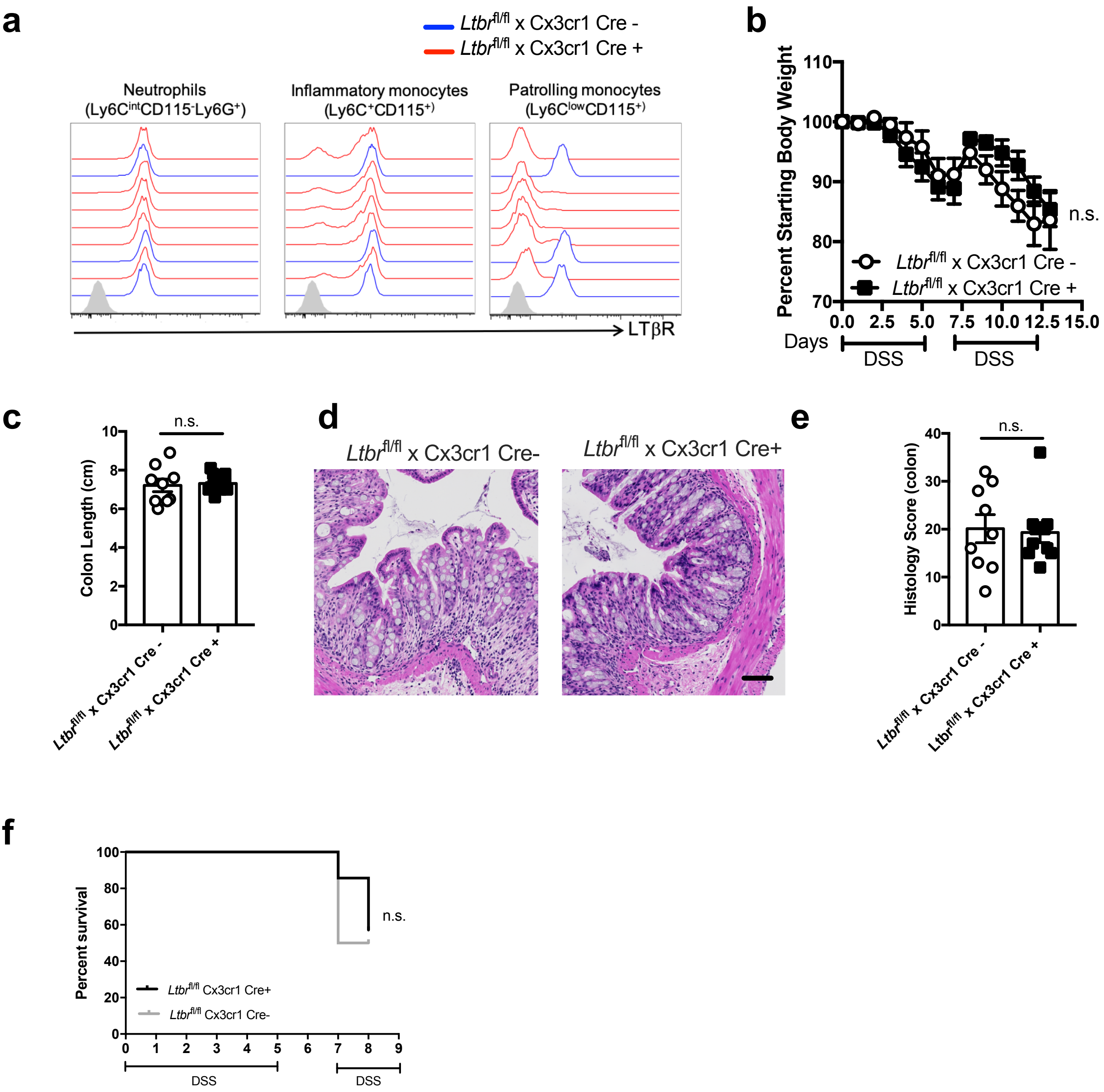


**Figure S1** Colitis is not exacerbated in mice with inducible Cx3cr1-Cre mediated deletion of LTβR Deletion of *Ltbr* was induced by i.p. tamoxifen treatment and colitis was induced in *Ltbr*$^{fl/fl}$ x Cx3cr1-Cre ERT mice and control littermates by cycles of 2.5 % DSS in drinking water for 5 days with 2 days break. **a** Representative histograms of LTβR expression in myeloid populations from the blood after Cx3cr1-Cre mediated deletion. Grey: isotype control. **b** Weight loss was monitored daily as % starting body weight. **c** Colon length on day 13. D) H&E stained representative sections of distal colon. Scale bar, 100 m **e** Blinded total histological score. **f** Kaplan-Meier survival plot of the indicated genotypes. Data represent two independent experiments (**a**, **d**, **f**) or are combined from 2 independent experiments (**b**, **c**, **e**). Error bars represent S.E.M.

# Supplemental Figure 2

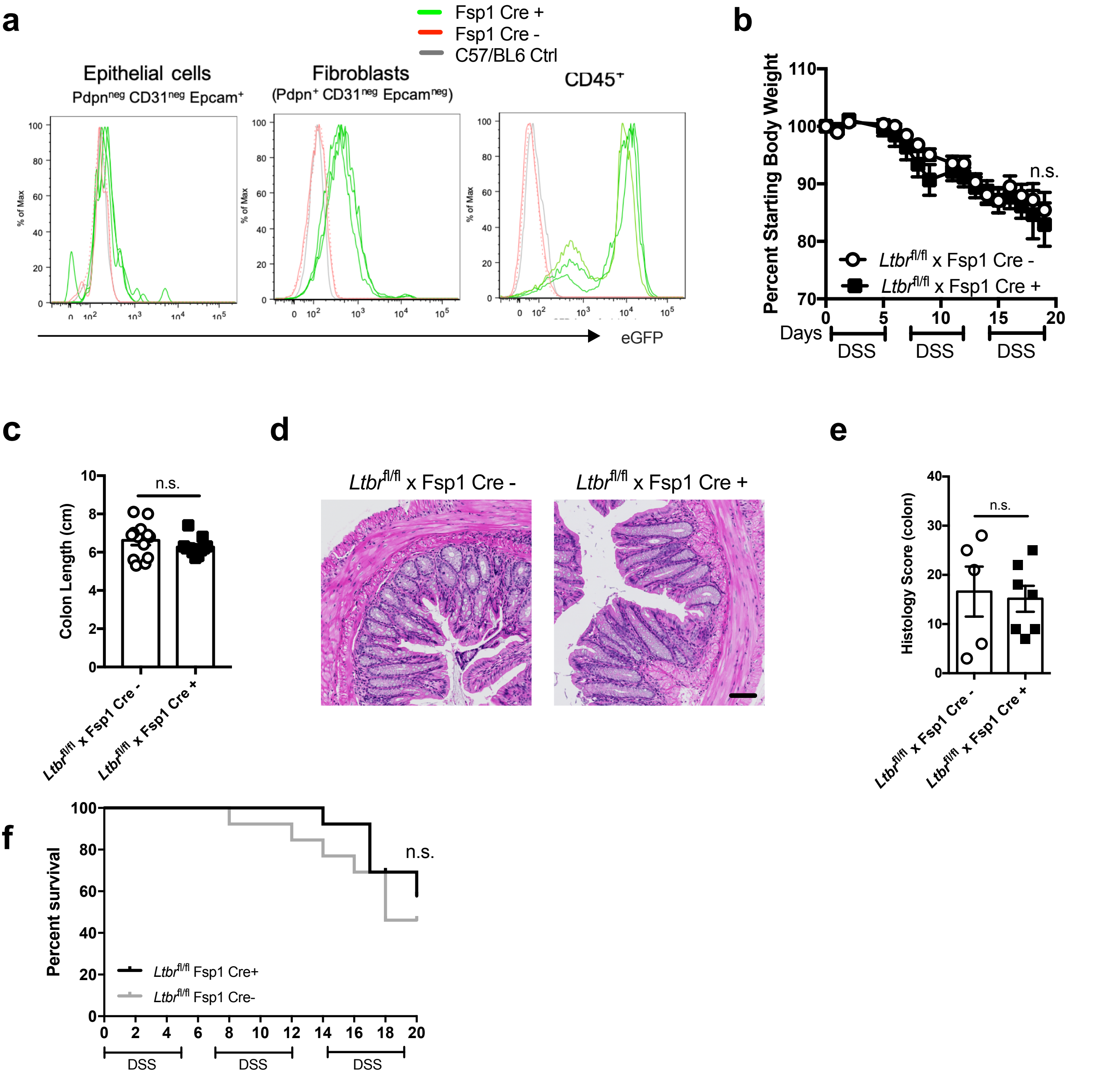


**Figure S2** Colitis is not exacerbated in chimeric mice with Fsp1-Cre mediated deletion of LTβR Colitis was induced in *Ltbr*$^{fl/fl}$ x Fsp1-Cre and control littermate recipients, reconstituted with WT hematopoietic cells to eliminate any off-target deletion effects of LTβR in hematopoietic cells. Cycles of 2.5 % DSS in drinking water were administered for 5 days with 2 days break and disease progression was monitored. **a** Representative histograms of eGFP reporter expression of Fsp1 activity in colonic cells from Fsp1-Cre+ mice showing Cre deletion in fibroblasts with significant off-target deletion in hematopoietic cells. **b** Weight loss was monitored daily as % starting body weight. **c** Colon length on day 19. **d** H&E stained example sections of distal colon with **e** blinded total histological score. **f** Kaplan-Meier survival plot of the indicated genotypes. Data represent 2 independent experiments (**a**, **d**, **e**) or are combined from two experiments (**b**, **c**, **f**). Error bars represent S.E.M.; Scale bar, 100 m.

# Supplemental Figure 3

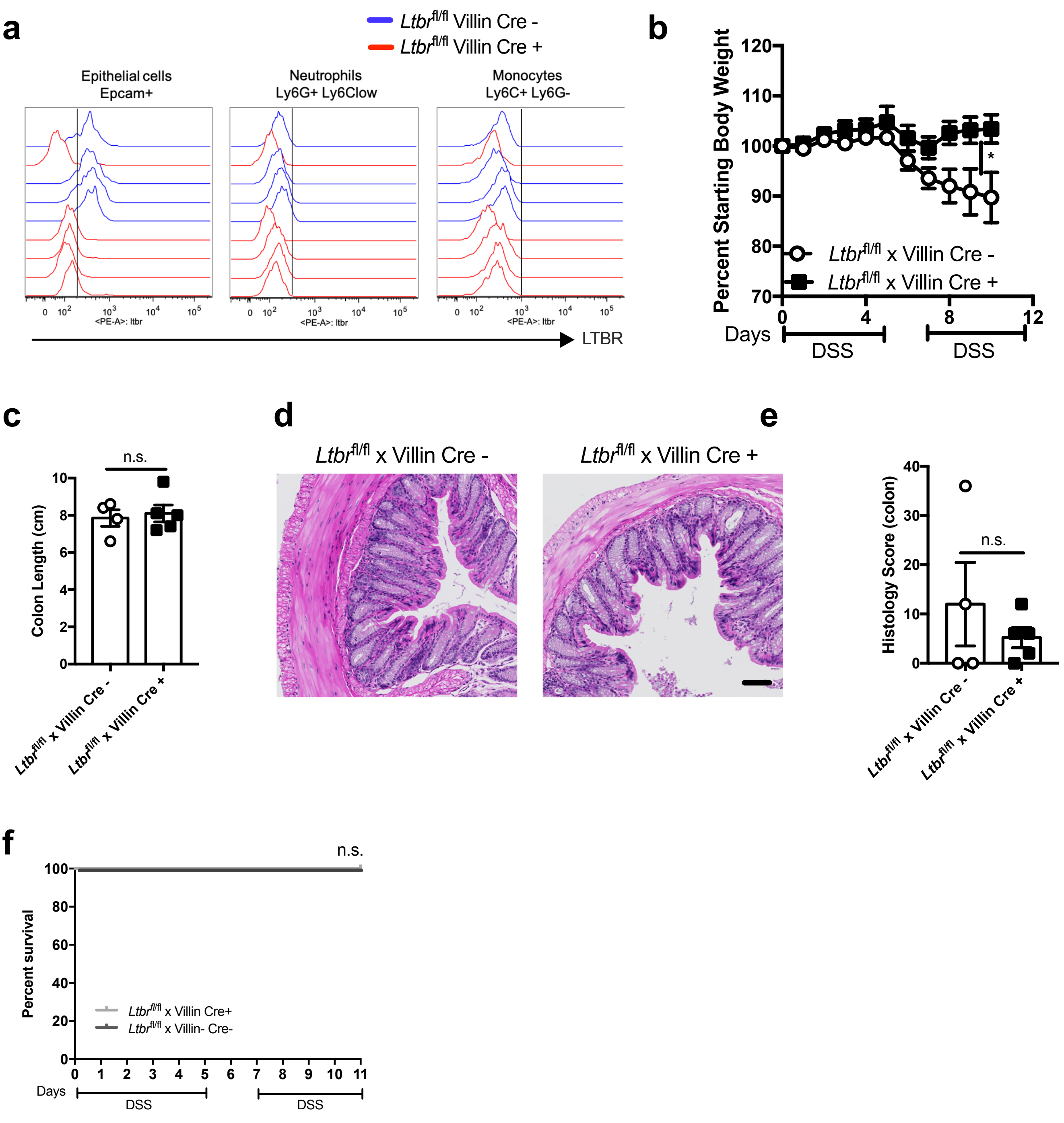


**Figure S3** Colitis is not exacerbated in mice with Villin-Cre mediated deletion of LTβR

Colitis was induced in *Ltbr*$^{fl/fl}$ x Villin-Cre and control littermates by cycles of 2.5 % DSS in drinking water for 5 days with 2 days break and disease progression was monitored. **a** Representative histograms of LTβR expression in indicated cell populations from colon of each genotype. **b** Weight loss was monitored daily as % starting body weight. **c** Colon length measured on day 10. **d** H&E stained example sections of distal colon with **e** blinded total histological score. **f** Kaplan-Meier survival plot of the indicated genotypes. n=4-5mice/group. Data represent one of two independent experiments. Error bars represent S.E.M.; Scale bar, 100 m.

# Supplemental Figure 4

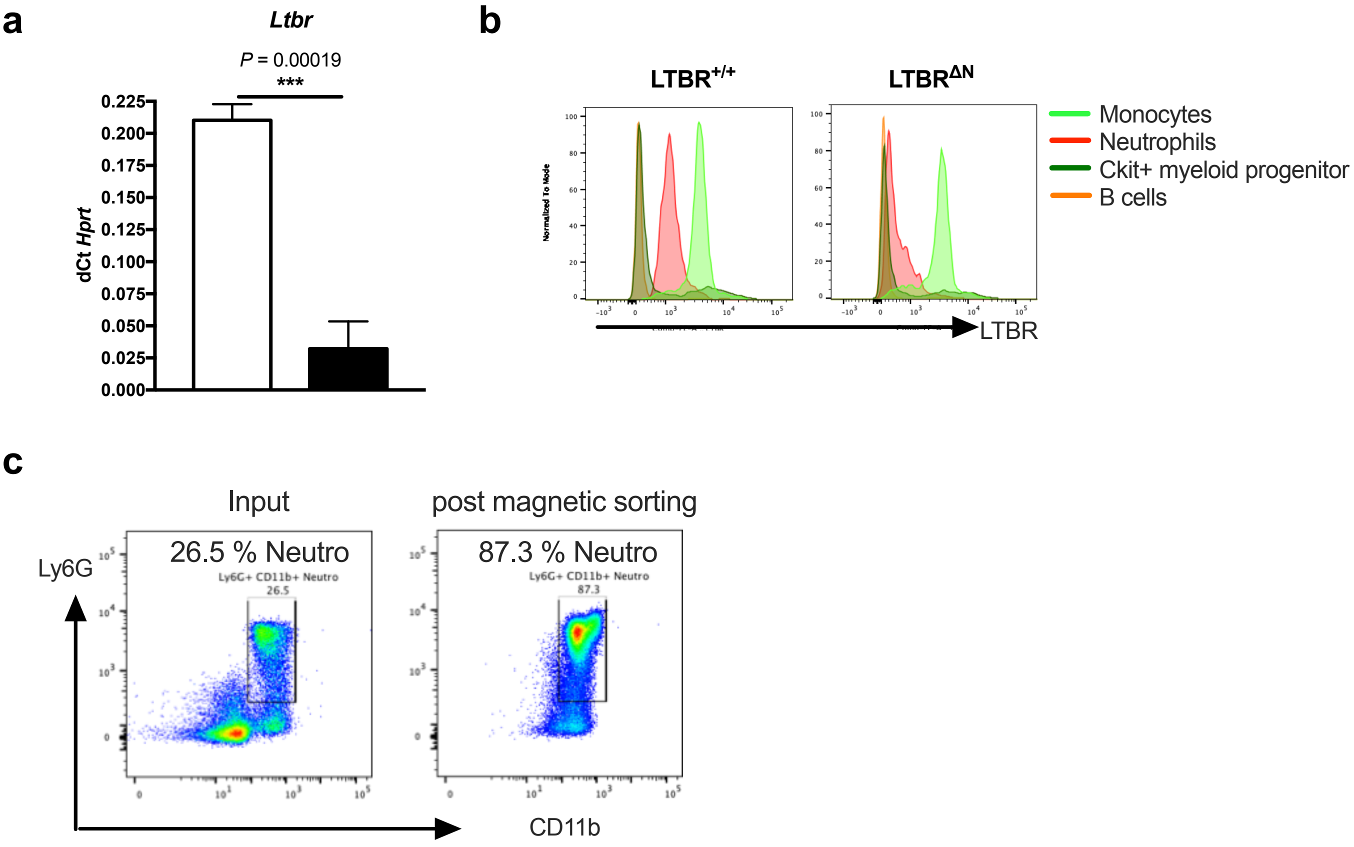


**Figure S4** Deletion efficacy of neutrophil LTβR expression in LTβR$^{\Delta N}$ mice.

**a** Neutrophils were isolated from blood of *Ltbr*$^{fl/fl}$ x Mrp8-Cre (LTβR$^{\Delta N}$) mice and gene expression was measured by qPCR. **b** Representative histograms of LTβR expression in monocytes, neutrophils, myeloid progenitors, and B cells in control (left) or LTβR$^{\Delta N}$ mice (right) (n=5). **c** Sort purity post EasySep™ magnetic enrichment (STEMCELL) was controlled by flow cytometry after gating on live, CD45+ cells. Data represent one of three independent experiments. Error bars represent S.E.M.

# Supplemental Figure 5

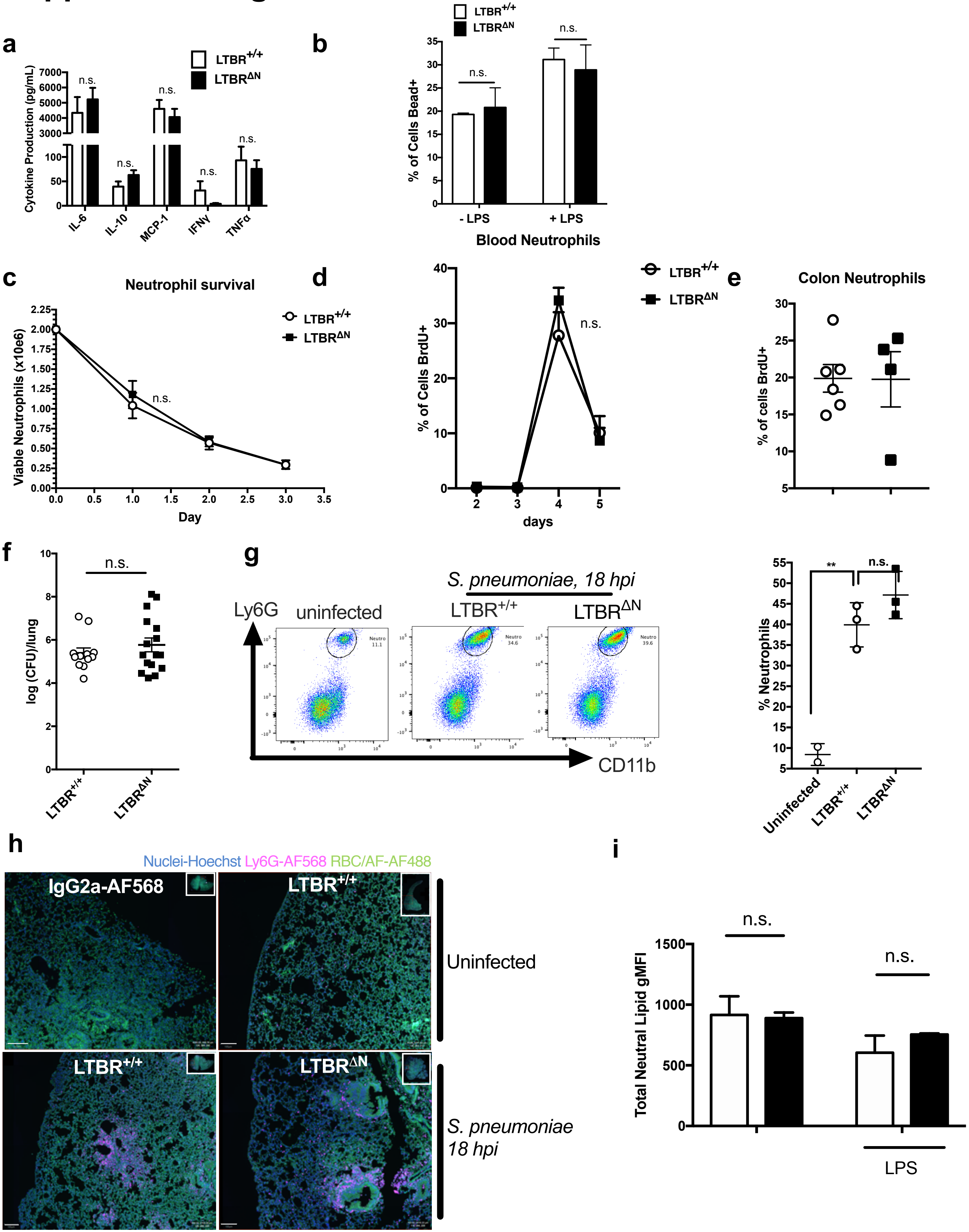

**Figure S5** Inflammatory cytokines and neutrophil function in LTβR$^{\Delta N}$ mice.

**a** Cytokine content in colon explant culture supernatant was measured by ELISA from punch biopsies on day 5 of DSS induced colitis. **b-e** Neutrophils were isolated from BM of *Ltbr*$^{fl/fl}$ x Mrp8-Cre (LTβR$^{\Delta N}$) and control littermates and assessed functionally. **b** Neutrophils were isolated by flow-cytometric sorting and phagocytosis of pHrodo$^{TM}$ labelled *E. coli* Bio-Particles was measured at baseline and in response to 100 ng/ ml LPS. **c** Survival time course of FACS-sorted neutrophils by trypan-blue exclusion in the absence of cytokines. **d** BrdU (2 mg/mouse) was injected i.p. on day 0 and the kinetics of appearance and disappearance of BrdU$^{+}$ neutrophils in the blood was tracked over 5 days. **e** The appearance of BrdU+ colon neutrophils was quantified by flow cytometry on day 7 of DSS treatment. **f** Mice were infected with *S. pneumoniae* strain URF918 retropharyngeally on day 0 and lung CFU was quantified 1 day later. **g** Representative histograms (left) and quantification (right) of neutrophil recruitment into lung was quantified 18 hours post *S. pneumoniae* infection by flow cytometry. **h** Immunofluorescent micrograph representation of Ly6G+ neutrophil recruitment (purple) into lung tissue at 18 hours post S. pneumoniae. **i** Neutral lipid content was measured by Bodipy 493/503 fluorescence intensity and analyzed by flow cytometry at baseline and in response to LPS (100 ng/ml). n.s., not significant. Error bars represent S.E.M. Data represent two or more independent experiments (**a-e, g-h**), or are combined from three experiments (**f**).

# Supplemental Figure 6

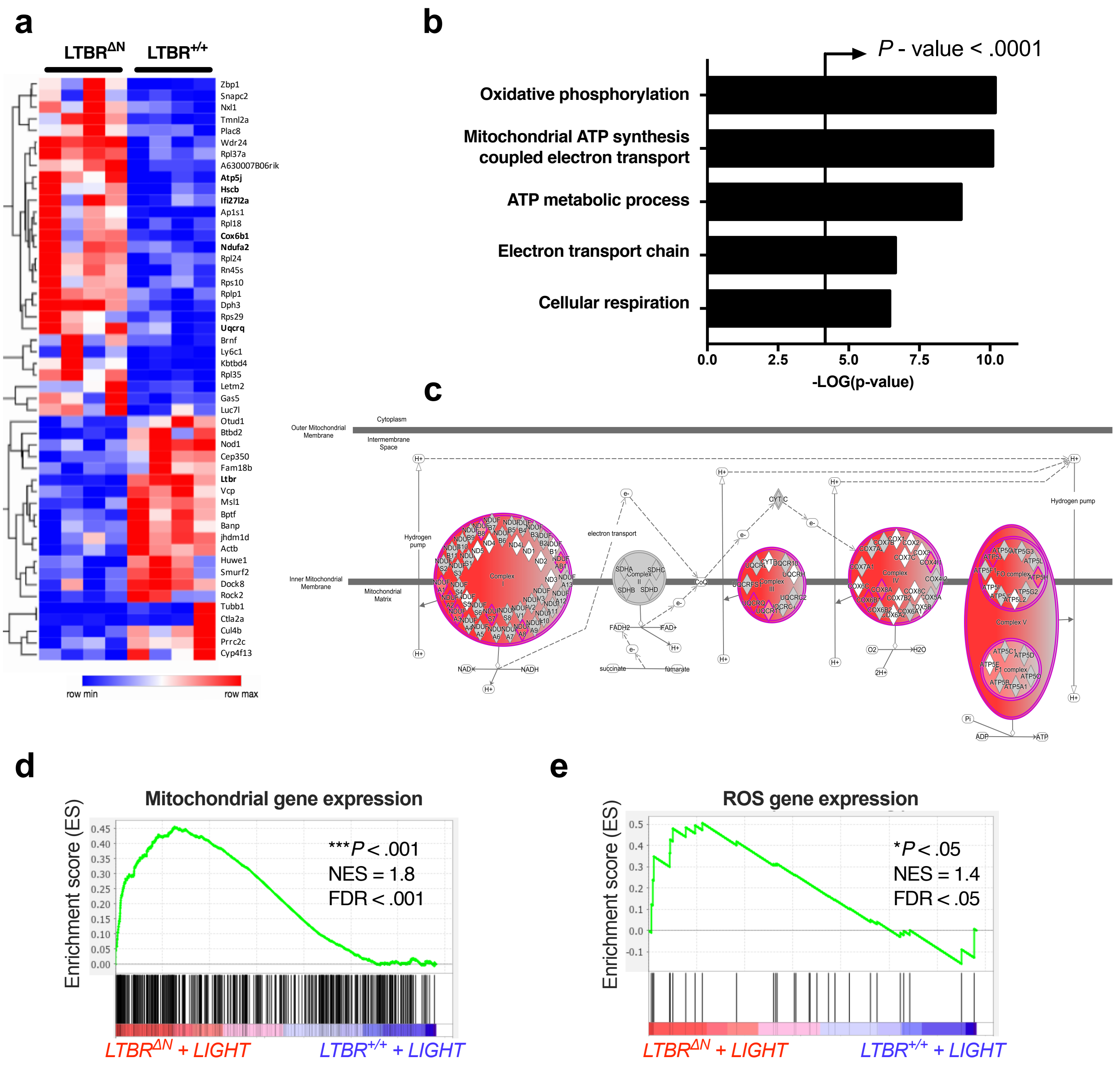


**Figure S6.** LIGHT-mediated suppression of genes associated with mitochondrial function and ROS requires neutrophil expression of LTβR.

RNA-Seq analysis of sorted blood neutrophils from LTβR$^{\Delta N}$ mice and littermate controls stimulated with a DSS-associated cytokine cocktail and recombinant LIGHT. **a** The top 50 most differentially expressed genes with respect to p-value. Bold gene names are associated with mitochondrial and ROS pathways. **b** Gene expression pathways upregulated in the absence of LTβR. **c** Ingenuity Pathway Analysis identified oxidative phosphorylation as a pathway highly upregulated in the absence of LIGHT stimulation. Significantly upregulated genes and complexes are indicated in red. **d-e** GSEA of the comparison of LTβR$^{\Delta N}$ mice and littermate controls. **d** GSEA for genes associated with mitochondrial function from the molecular signature database list GO_mitochondrial_gene_expression. NES = 1.8; FDR < .001. **e** GSEA for genes associated with cellular ROS from the molecular signature database list Houstis_ROS. NES = 1.4; FDR < .05. GEO accession number GSE150243.

# Supplemental Figure 7

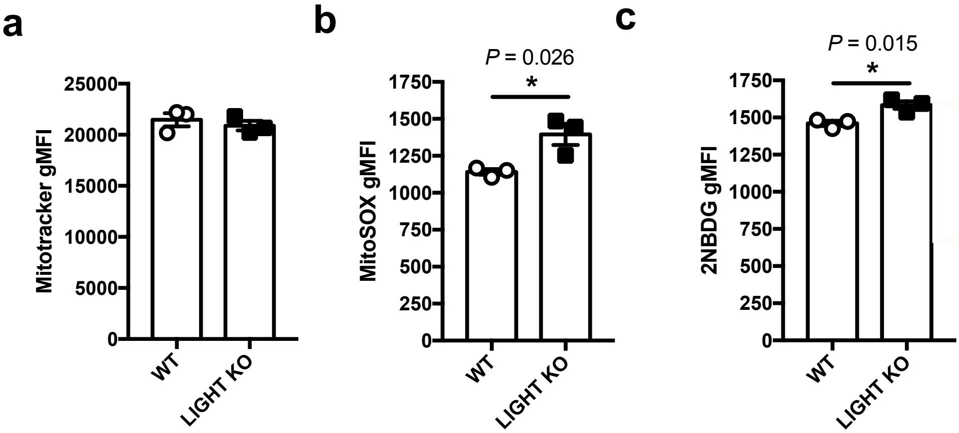


**Figure S7** LIGHT signaling inhibits neutrophil mitochondrial ROS and glycolysis

Neutrophils were isolated from BM of LIGHT$^{-/-}$ mice and control littermates and metabolic parameters were measured. **a** Mitochondrial mass was measured by Mitotracker intensity using flow cytometry. **b** Mitochondrial ROS was measured by MitoSox fluorescence intensity by flow cytometry. **c** The uptake of the fluorescent glucose analogue 2NBDG was quantified by flow cytometry as gMFI. Data from 3 mice/group, representative of 3 independent experiments. Error bars represent S.E.M.

# Supplemental Figure 8

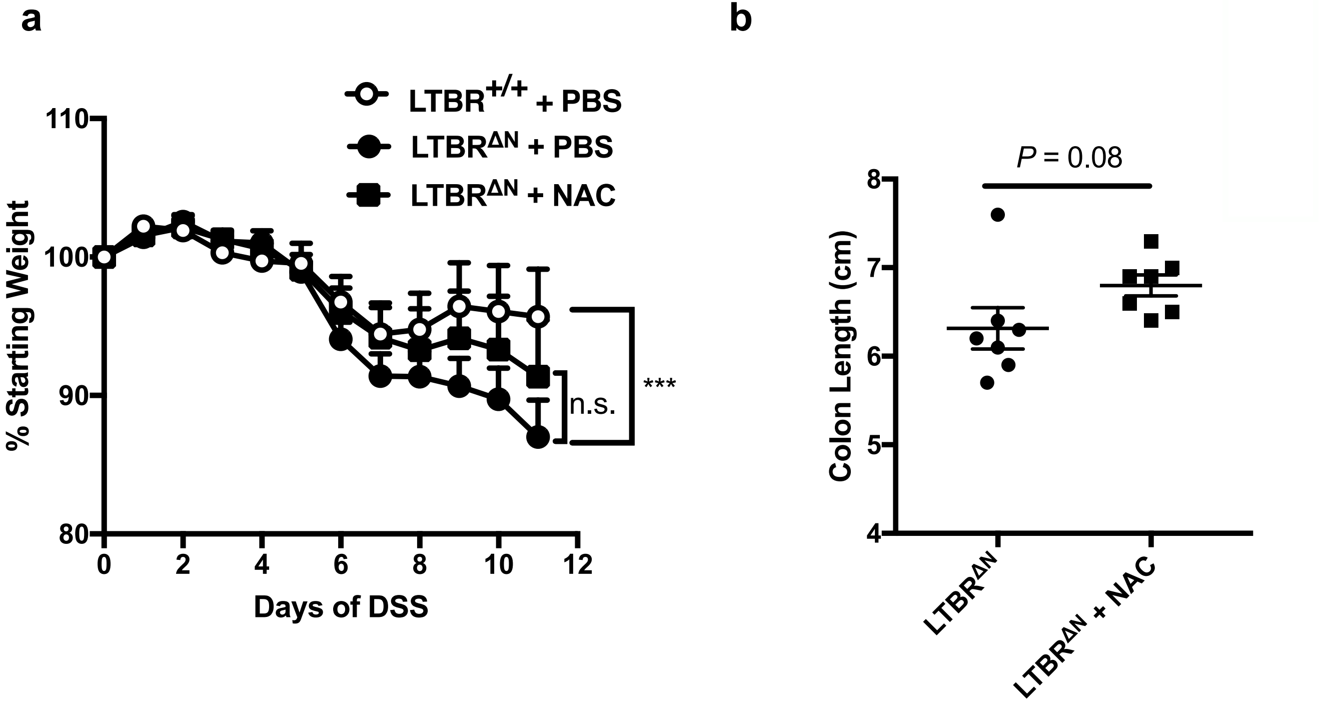


**Figure S8** N-acetyl cysteine treatment did not reverse exacerbated disease in LTBR mice

Colitis was induced in *Ltbr*$^{fl/fl}$ x Mrp8-Cre (LTβR$^{\Delta N}$) and control littermates by 2.5 % DSS. N-Acetyl-Cysteine (150 mg/kg) or PBS was administered daily by i.p. injection and disease parameters were measured. **a** weight loss was monitored daily as % starting body weight. **b** Colon length on day 11. Data represents 3 independent experiments (**a**) or is combined from 2 independent experiments (**b**). Error bars represent S.E.M.

**Table 1 List of genes that are among top50 differentially regulated by LIGHT and with redox or mitochondrial Gene Ontology**

| | | |
|---|---|---|
| Atox1 | Antioxidant 1 Copper Chaperone | Redox |
| Atp5e | Mitochondrial ATP Synthase F1 Subunit Epsilon | Mitochondrial |
| Cox8a | Mitochondrial Cytochrome C Oxidase Subunit 8A | Mitochondrial |
| CybA | Superoxide-Generating NADPH Oxidase Light Chain Subunit | Redox |
| Atp5j | ATP Synthase, H+ Transporting, Mitochondrial F0 | Mitochondrial |
| Tomm7 | Translocase Of Outer Mitochondrial Membrane 7 | Mitochondrial |
| Cox4i1 | Cytochrome C Oxidase Subunit 4I1 | Mitochondrial |
| Cox6b1 | Cytochrome C Oxidase Subunit 6B1 | Mitochondrial |
| Ndufa3 | NADH:Ubiquinone Oxidoreductase Subunit A3 | Mitochondrial |
| Taldo1 | Transaldolase 1 Sulfhydryl protector | Redox |
| Gpx4 | Glutathione Peroxidase 4 | Redox |

**Supplementary Methodology**

**Cell isolation**

Bone marrow neutrophils were obtained by crushing tibia and femur with a mortar and pistil in RPMI1640 supplemented with 5% FBS. The suspension was filtered through a 70um mesh washed and pelleted. Following red blood cell lysis, non-granulocytic cells were immunolabelled with a mixture of antibodies according to the Neutrophil magnetic bead negative selection kit (Stem Cell Technologies), or labelled with CD11b and Ly6G antibodies for direct, positive sorting via flow cytometry. Purity of obtained neutrophils was controlled post-sort and was typically >90% (negative selection) or >95% (positive selection).

**RNA-Seq data acquisition and analysis**

Neutrophils were isolated from the blood of 8-week-old LTβR$^{\Delta N}$ mice, and littermate controls, using a magnetic bead negative selection kit (Stem Cell Technologies). Following isolation, cells were stimulated for 4 hours with cytokines (TNF [100 ng/ml; R&D systems], IL-6 [50 ng/ml; Peprotech] and GM-CSF [100 ng/ml; R&D systems]) in the presence or absence of soluble LIGHT stimulation (500 ng/ml, R&D systems). Cells were lysed in Trizol LS (Thermo Fisher). RNA extraction, reverse transcription, cDNA amplification, fragmentation, additional PCR amplification, final library purification, and quality control were performed as previously described[1]. Sequencing was performed according to a single-end strategy using the HiSeq2500 sequencer (Illumina). Samples that failed quality control standards were eliminated from further analysis[1]. The single-end reads that passed Illumina filters were filtered for reads aligning to tRNA, rRNA, adapter sequences, and spike-in controls. The reads were then aligned to UCSC mm9 reference genome using TopHat (v 1.4.1). DUST scores were calculated with PRINSEQ Lite (v 0.20.3) and low-complexity reads (DUST > 4) were removed from the BAM files. Read counts to each genomic feature were obtained with the Ht-Seq-count program (v 0.6.0) and imported to R/Bioconductor package DESeq2 to identify differentially expressed genes among samples. *P*-values for differential expression were calculated using binomial test and adjusted for multiple test correction using the

Benjamini-Hochberg procedure. We considered genes differentially expressed if the adjusted *P*-value was <0.1 and the fold change in gene expression was >1.5.

For pathway analysis, the database at http://toppgene.cchmc.org/ was employed. Briefly, genes upregulated in the noted groups with a nominal *P*-value < 0.05 were applied to analysis. For pathway visualization, Ingenuity Pathway Analysis (Qiagen) was used. For Gene Set Enrichment Analysis (GSEA) data were analyzed using the pre-ranked GSEA algorithm (Broad Institute and University of California). Molecular Signatures Database (MSigDB) gene lists for mitochondrial genes and ROS genes were applied to a ranked list of all genes in the noted comparisons and run in the GSEA graphical user interface.

**Flow cytometry**

Fluorochrome-conjugated monoclonal antibodies were purchased from eBioscience, BD Bioscience, or BioLegend. Antibodies with clone indicated in parentheses: anti-mouse CD45 (30-F11); anti-mouse Ly6g (1A8); anti-mouse CD11b (M1/70); anti-mouse CD62L (MEL-14); anti-mouse LTβR (5G11); anti-mouse CD117/c-kit (2B8); anti-mouse CD45R/B220 (RA3-6B2); anti-mouse CD11c (N418). Metabolic cytometry assays have been described previously[2]. Briefly, cells were stained with MitoTracker Green or MitoTracker deep-Red (Life Technologies) at 100 nM concentration; for quantification of mitochondrial superoxide, cells were stained with MitoSox Red (ThermoFisher) at 5µM; for glucose uptake measurements, cells were incubated in media containing 5 µg/ml 2-(*N*-(7-Nitrobenz-2-oxa-1,3-diazol-4-yl)Amino)-2-Deoxyglucose (2-NBDG); for lipid droplet quantification, cells were incubated in media containing 1 µg/ml Bodipy. Incubation periods for dye or metabolite uptake depend on tissue and required fluorescence intensity, but did not exceed 45 minutes. Data were acquired using Fortessa or LSR II flow cytometers (BD Biosciences) and analyzed with FlowJo software. Metabolic marker fluorescence intensity depends on the instrument type and laser intensity, and therefore does not allow inter-experiment comparisons.

**Compounds**

N-acetyl-L-cysteine (NAC) ≥99% (TLC) was purchased from Sigma, Metformin (3-(diaminomethylidene)-1,1-dimethylguanidine;hydrochloride) was from Tocris Bioscience.

**Metabolic flux analysis**

3 × $10^5$ freshly isolated Ly6G$^+$ BM neutrophils were obtained by flow-cytometric sorting using CD11b and Ly6G. Post-sort purity was >95%. Neutrophils were washed twice in the assay medium consisting of bicarbonate-free DMEM base (Sigma, D-5030-1L), 1.85 g/L NaCl and 6 mg/ml Phenol Red supplemented with 5 mM HEPES-Na, pH7.6, 10 mM glucose, 10 mM pyruvate and 4 mM glutamine, then attached in the assay medium to a XF plate coated with Cell-Tak (Sigma) by spinning at 500 *g* for 5min. Cells were rested for 20min in a non-$CO_2$ incubator at 37°C before analysis. Mitochondrial stress test was performed with all rates read twice, with 30 sec of mixing followed by 2min data acquisition. The initial reading of the basal rates was followed by injection of 2 µg/ml oligomycin to stop mitochondrial ATP synthesis and force compensatory stimulation of glycolysis. Subsequently, two sequential injections of 300 µM uncoupler 2,4-dinitrophenol (DNP) were made to ensure attaining the maximal OCR[3]. The run was terminated with the injection of 2 µM myxothiazol, a respiratory inhibitor targeting Complex III to control for non-mitochondrial background rate[4]. The basal rates reflect cellular ATP turnover and, thus, served as a measure of cellular energy demand; maximal uncoupler-stimulated OCR and oligomycin-induced ECAR served as the measures of cellular respiratory and glycolytic capacities, respectively.

**Electron Microscopy**

Incubation of the cell pellet with 1 % $OsO_4$ in buffer for 30 min was followed by three washing steps with $dH_2O$. The sample was incubated with 2 % uranyl acetate, rinsed, and dehydrated in EtOH. Embedded cells were then placed in a 60°C oven overnight. Sections

were cut on a Leica Ultracut microtome and collected on copper grids. Sections were stained with 2 % uranyl acetate in $dH_2O$ and Sato Lead. The TEM evaluation was carried out on the JEOL-1200 EX microscope.

**Supplementary references**


1. Rosales SL, Liang S, Engel I, et al. A Sensitive and Integrated Approach to Profile Messenger RNA from Samples with Low Cell Numbers. Methods Mol. Biol. 2018;1799:275–302.

2. Puleston DJ, Zhang H, Powell TJ, et al. Autophagy is a critical regulator of memory CD8(+) T cell formation. Elife 2014;3:2516.

3. Terada H. Uncouplers of oxidative phosphorylation. Environ. Health Perspect. 1990;87:213–218.

4. Ksenzenko M, Konstantinov AA, Khomutov GB, et al. Effect of electron transfer inhibitors on superoxide generation in the cytochrome bc1 site of the mitochondrial respiratory chain. FEBS Lett. 1983;155:19–24.
